# Neurotechnology for enhancing human operation of robotic and semi-autonomous systems

**DOI:** 10.3389/frobt.2025.1491494

**Published:** 2025-05-23

**Authors:** William J. Tyler, Anusha Adavikottu, Christian Lopez Blanco, Archana Mysore, Christopher Blais, Marco Santello, Avinash Unnikrishnan

**Affiliations:** ^1^ Department of Biomedical Engineering, University of Alabama at Birmingham, Birmingham, AL, United States; ^2^ Center for Neuroengineering and Brain Computer Interfaces, Birmingham, AL, United States; ^3^ Department of Civil, Construction, and Environmental Engineering, Birmingham, AL, United States; ^4^ Department of Neurosurgery, UAB School of Medicine, Birmingham, AL, United States; ^5^ School of Biological and Health Systems Engineering, Arizona State University, Tempe, AL, United States

**Keywords:** human-machine, neurotechnology, neuromodulation, BCI, cognition, teleoperations, attention

## Abstract

Human operators of remote and semi-autonomous systems must have a high level of executive function to safely and efficiently conduct operations. These operators face unique cognitive challenges when monitoring and controlling robotic machines, such as vehicles, drones, and construction equipment. The development of safe and experienced human operators of remote machines requires structured training and credentialing programs. This review critically evaluates the potential for incorporating neurotechnology into remote systems operator training and work to enhance human-machine interactions, performance, and safety. Recent evidence demonstrating that different noninvasive neuromodulation and neurofeedback methods can improve critical executive functions such as attention, learning, memory, and cognitive control is reviewed. We further describe how these approaches can be used to improve training outcomes, as well as teleoperator vigilance and decision-making. We also describe how neuromodulation can help remote operators during complex or high-risk tasks by mitigating impulsive decision-making and cognitive errors. While our review advocates for incorporating neurotechnology into remote operator training programs, continued research is required to evaluate the how these approaches will impact industrial safety and workforce readiness.

## Introduction

**FIGURE 1:**
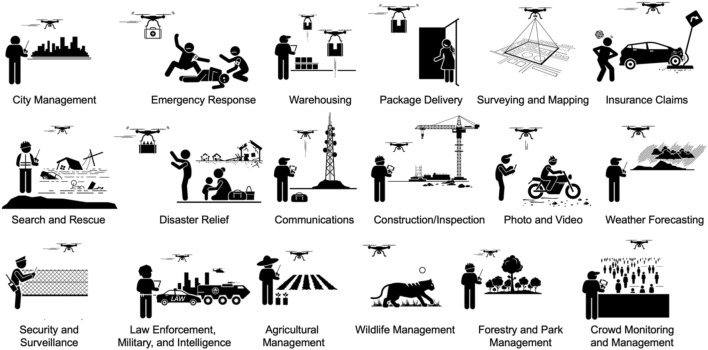
Commercial and industrial utility of remotely piloted drones. The iconographic illustrations depict some example applications of sUAS/UAVs that are creating an impact on many different industries. The development and deployment of different types of sensors and edge computing methods has led to a growing adoption of drones and other remotely operated robotic systems for diverse industrial applications as illustrated. This broadening use of semi-autonomous drones and robotic systems in commercial, security, and defense applications means workforce and talent development efforts will also grow to include the establishment of specific training programs and certifications. Some training programs may incorporate modern neurotechnology methods to enhance remote operator cognition and performance.

The growth and adoption of robotics, artificial intelligence (AI), and industrial automation is changing the nature of human-machine interactions (HMI). Industrial sectors like shipping, excavation, road making, construction, and commercial truck driving are becoming increasingly reliant on remotely operated and semi-autonomous systems, which requires training a new generation of skilled operators. These remote and semi-autonomous systems require trained personnel, who are capable of monitoring complex sensor information, interpreting real-time data, and making rapid decisions in dynamic environments. The use of small unmanned aerial systems (sUAS) or unmanned aerial vehicles (UAVs) is changing the workforce across many industries (Tezza and Andujar, 2019; Floreano and Wood, 2015; Rao et al., 2016; Merkert and Bushell, 2020) (Figure 1). Similarly, the past decade has seen immense growth in the use of semi-autonomous mining equipment, remotely operated agricultural machines, and robotic cargo handling systems. Collectively, these remotely operated machines are revolutionizing industrial efficiency, productivity, and safety (Tezza and Andujar, 2019; Schönböck et al., 2022; Wanasinghe et al., 2021; Pizoń and Gola, 2023; Soori et al., 2023). These modern systems introduce new cognitive challenges for HMI’s and our workforce. In the present review, we evaluate literature suggesting these challenges can be addressed by incorporating neurotechnology into advanced training and simulation methods.

**FIGURE 2:**
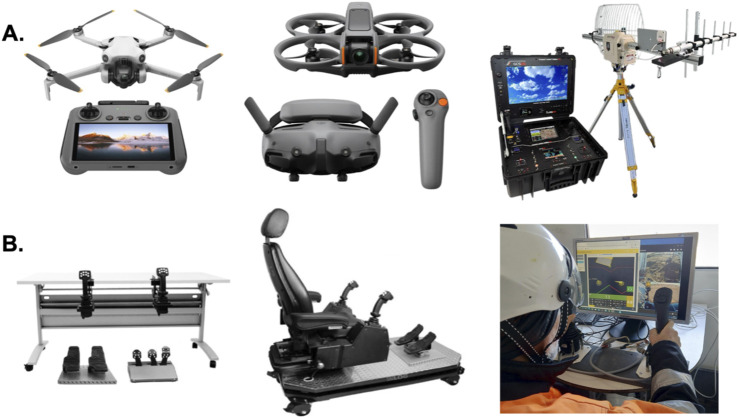
Human interfaces for training and operation of drones and remotely controlled machines. **(A)** Modern aerial drones utilize different types of digital human control interfaces, such as joysticks with integrated screens (left) to heads-up displays with integrated motion controllers (middle). Some drone and remote vehicle interfaces implement analog transmission of control, video, and sensor data with directional RF antennas, displays, and joysticks (right). The displays and controllers in these systems convey important information to the operator from optical sensors, gyroscopes and accelerometers, environmental sensors, positioning systems, and many other types of sensors and actuators. **(B)** Simulator control systems can prepare remote operators for real-world operations. Modern simulator equipment may include joysticks and foot pedals (left) or operator’s chairs (middle) that can be connected to computers, displays, and virtual reality systems to replicate vehicle and environmental situations. These simulators have proven indispensable to the cognitive and sensorimotor training of operators for complex robotic tasks like remote excavation (right). The left and middle image were reproduced from reference (Burk et al., 2023) and the right image was reproduced from reference (Hiltunen et al., 2023).

Remote operation of drones and robotic machinery can be a cognitively demanding task that requires distributed attention, efficient decision making, and physical multi-tasking under a high psychological stress load (Cross and Ramsey, 2021; Ljungblad et al., 2021; Lee et al., 2022). Remote operators face significant cognitive and sensorimotor challenges that differ from traditional vehicle operation. They must continuously process and interpret multi-modal sensory data from cameras, ultrasonic and laser range sensors, and other real-time data transmission. Due to various hardware, firmware, software, and human interface variables, the streaming and display of data can be wrought with timing delays or inconsistencies challenging the operator. For example, the reliance on indirect visual input rather than direct line-of-sight decreases situational awareness, which requires operators to develop strong spatial reasoning and cognitive control skills. Other performance burdens arise due to the complexity and timing differences in multi-sensory integration and processing demands placed upon teleoperators. For example, human processing of digitized video streams or visual feeds may not align with their endogenous proprioceptive, cognitive, or real-world visual processing cues. Furthermore, fine motor control through joysticks or haptic interfaces requires sustained attention due to the precise coordination between cognitive decision making and sensorimotor execution (Figure 2). This often leads to operator mental and physical fatigue over prolonged operations. Operators must also contend with unexpected environmental changes, system failures, and the psychological strain of high-stakes decision-making. Thus, cognitive endurance and robust executive function are essential for the optimal performance of remote operators.

The robotics industry is undergoing an evolution in which artificial systems are beginning to adapt to and learn human operator variables. To advance control and command systems capable of cognitive co-evolution, neurotechnology can deliver methods and devices centered on helping the human operator learn and adapt to these robotic and semi-autonomous systems. Developing solutions intended to enhance remote operator training and performance represents an immense opportunity for incorporating neurotechnology to improve learning, cognition, and stress responses for human operators of semi-autonomous robotic machines and systems. Training systems and programs have recently evolved to include high-fidelity simulators that provide realistic operational environments, which reflect the operational experiences and complexities of real-world tasks (Figure 2). Training of remote operators using realistic simulators improves safety and reduce costs across many industries where humans monitor and control the work of semi-autonomous machines (Schönböck et al., 2022; Lee et al., 2022; Chirgwin, 2021; Adami et al., 2021; Han et al., 2023; Shringi et al., 2022; Warren et al., 2023; Xu and Zheng, 2021). Government and industry regulatory bodies including the Occupational Health and Safety Administration (OSHA), Department of Transportation (DOT), Federal Aviation Administration (FAA), US Department of Agriculture (USDA), and many others have engaged in the development of innovative training approaches to ensure workforce readiness as industries increasingly rely on HMI’s and remotely operated machines (Adami et al., 2021; Burk et al., 2023; Goode et al., 2013; Veitch and Andreas Alsos, 2022).

Like the growth of the robotics industry, the field of neuroengineering has also experienced recent growth. There have been many advances in the development and commercialization of methods and devices for sensing and modulating human brain activity, behavior, and cognition. Neuromodulation techniques like transcranial electrical stimulation (tES), transcranial focused ultrasound (tFUS) neuromodulation and transcutaneous vagus nerve stimulation (tVNS) offer promising solutions to optimize training and performance outcomes for remote operators. As described below, these methods work by enhancing neuroplasticity, which helps operators learn better and more easily adapt to complex control systems. Recent studies demonstrate that tES and tVNS can improve resilience, decision-making, multimodal attention, vigilance, and cognitive flexibility (Choe et al., 2016; McIntire et al., 2023; Feltman and Kelley, 2024; Hemmerich et al., 2024). By integrating these noninvasive brain stimulation methods into training programs, organizations can accelerate skill acquisition and optimize cognitive function, leading to a more capable and adaptable workforce. Furthermore, the incorporation of neurofeedback and AI-driven sensor enhancements into HMI training can improve situational awareness and real-time cognitive performance. Using electroencephalography (EEG) or functional near-infrared spectroscopy (fNIRS), neurofeedback approaches can provide operators with real-time monitoring of cognitive states while helping them regulate attention and optimize decision-making (Faller et al., 2019; Kim et al., 2014; Duan et al., 2019; Khan and Hong, 2017; Deng et al., 2023). With drones and other autonomous machines becoming an integral part of everyday industrial and commercial operations, investment in neurotechnology-driven operator training and human-machine optimization will be crucial in shaping the future of autonomous systems management.

### Synthetic environments and realistic training programs for enhancing remote operator performance

The increasing reliance on remote, robotic, and semi-autonomous systems across industries requires the development of structured training programs to ensure operators acquire the necessary cognitive and technical skills to perform safely and efficiently. New technologies enable the delivery of realistic training programs in synthetic environments designed to enhance a remote operator’s sensorimotor skills, situational awareness, executive function, and decision-making while cooperatively working with robotic machinery (Figure 2B). Unambiguously, a wealth of literature shows realistic training simulations enhance workforce preparedness across multiple industries, including UAV operations, excavation, forestry, mining, and robotic-assisted surgery (Schönböck et al., 2022; Adami et al., 2021; Shringi et al., 2022; Burk et al., 2023; Liu et al., 2013; Sridhar et al., 2017). By utilizing simulation-based training and virtual reality (VR) environments, these programs provide hands-on experience in a controlled setting, allowing trainees to develop critical competencies without exposing them to real-world hazards.

High-fidelity simulators recreate real-world operational scenarios with precise control interfaces and environmental conditions, allowing operators to practice responding to emergency situations, system failures, and high-pressure decision-making tasks (Figure 2). For example, UAV operator performance is enhanced following training in simulated flight environments to develop precision in navigating airspace, reacting to unexpected obstacles, and coordinating with autonomous flight systems (Schmidt et al., 2022; Sakib et al., 2021; Somerville et al., 2024). In mining, forestry, and excavation, for example, operators trained through realistic VR-based simulation programs demonstrate greater proficiency in navigating hazardous terrain, managing automated excavation equipment, and optimizing resource extraction processes compared to those trained using traditional methods. Similarly, robotic surgeons undergo rigorous simulator-based training before transitioning to live procedures, ensuring proficiency in remote-controlled surgical techniques before working on actual patients (Sridhar et al., 2017; Moit et al., 2019; Mallela et al., 2022). One of the primary advantages of simulation-based training is its ability to improve operator safety and efficiency. By exposing trainees to high-risk scenarios in a risk-free environment, they are better equipped to handle real-world stressors when performing complex remote operations.

### Adaptive factors approaches to enhancing human-machine interactions

The effectiveness of realistic training programs can be further enhanced using adaptive technologies designed to enhance human-in-the-loop performance. Robotic and semi-autonomous machines do not experience physiological stress and are less prone to cognitive fatigue and failure than human operators. For example, by optimizing the cognitive workload of trainees during prolonged training implementing adaptive training logic and methods have been shown to enhance virtual F-16 cockpit training compared to non-adaptive VR methods (Aguilar Reyes et al., 2023). The use of haptics to provide tactile sensory input and somatosensory feedback related to texture, hydraulics, or force during virtual training has been shown to improve robotic teleoperator training outcomes and performance compared to control approaches not using haptics (Edmondson et al., 2012; Patel et al., 2022; Gani et al., 2022; Zhu et al., 2021; Yang et al., 2021; Xia et al., 2023). A recent study compared the influence of multi-modal sensory inputs on teleoperator performance. It was shown the use of stereoscopic 3D or VR displays improve task performance and accuracy by 40% compared to monocular displays and integration with vibrotactile and auditory feedback further improve performance (Triantafyllidis et al., 2020). Deeper investigations have studied the multisensory impact of encoding force with light, sound, and vibration on cognitive workload using EEG during robotic machine training (Haruna et al., 2021). Haruna and colleagues (2021) found that strategically positioned visual feedback encoding robotic force produced the most efficient haptic approach for reducing cognitive load or mental work during training (Haruna et al., 2021). Improving our physiological models of stress and mental workload can greatly enhance remote operator training, performance, and safety (Sakib et al., 2021). As further discussed below, neuroscience-guided approaches can further enable the reduction of cognitive fatigue by modulating arousal while distributing cognitive resources across different sensory (somatosensory, visual, and auditory cortex) and executive decision-making regions of the brain (prefrontal, temporoparietal, and cingulate cortex).

The use of haptic methods to improve teleoperator training and performance presents several interesting oppurtunities for advance the design and human factors elements of HMI’s. The somewhat recent development of soft, wearable sensors and actuators have led to the development of wearable haptic interfaces for improving the sensory and situational awareness of remote operators (Yin et al., 2021; Grasso et al., 2023; Aggravi et al., 2018). More specifically, several wearable embodiments like haptic sleeves, gloves, and shoes have been shown to enable neuromorphic control, enhanced environmental awareness, and overall improved teleoperator performance (Macchini et al., 2020; Thakur et al., 2024; D’Abbraccio et al., 2019). Efforts to engineer innovative haptic interfaces will make HMI’s more usable and reliable for integration into advanced training programs. For example, using soft, compliant electroactive hydrogels or polymers for sensing and actuation can improve the interface between human operators and hardware control systems (Xu et al., 2015). Stretchable hydraulic amplified actuators can be 3D printed and personalized to a user’s fingertips to enhancing cutaneous haptics without affecting dexterity (Grasso et al., 2023). State-of-the-art bioelectronic methods recently led to the engineering artificial ‘e-skin’ that can be used for neuromorphic robotic control and sensorimotor loop integration in HMI’s (Wang et al., 2023). Given observations that visual haptics can enhance operator performance while reducing cognitive load, the use of electroluminescent force sensors in tactile skin may represents an interesting approach for enhancing teleoperator HMI’s (Aggravi et al., 2018). The literature suggests that combining advanced bioelectronic interfaces with proven realistic training methods can significantly enhance the skill training, cognition, safety, and preparedness of drone, robotic, and remote machine operators.

### Noninvasive neuromodulation for enhancing human-machine cognition and teleoperator performance

Noninvasive neuromodulation approaches, such as transcranial magnetic stimulation (TMS), transcranial electrical stimulation (tES), transcranial focused ultrasound (tFUS), and transcutaneous vagus nerve stimulation (tVNS) have gained attention the past couple decades for their ability to treat various neurologic and psychiatric disorders (Tyler et al., 2017; Bhattacharya et al., 2022). These multimodal neuromodulation methods have also gained attention in consumer electronics, enterprise businesses, sports, and health industries for their ability to improve brain plasticity, enhance cognition, reduce stress, improve sleep, and optimize decision-making (Herrmann et al., 2013; Cinel et al., 2019; Grover et al., 2023; Colzato et al., 2017; Tyler, 2017; Antal et al., 2022). The noninvasive neuromodulation literature provides compelling evidence that these methods are safe for use in healthy humans suggesting they may be readily integrated into remote operator training programs (Tyler, 2017; Santarnecchi et al., 2015; Rossi et al., 2021; Kim et al., 2022; Antal et al., 2017). We are motivated to specifically evaluate wearable approaches with real-time modulation capabilities that can be easily incorporated into workforce training and performance programs. Thus, we restrict our further discussion of neuromodulation below to tES, tVNS, and tFUS methods. Presently, the electrical power and magnetic resonance image-guided neuronavigation recommendations for TMS makes it difficult to deploy in scalable training and performance enhancement solutions across industries.

**FIGURE 3:**
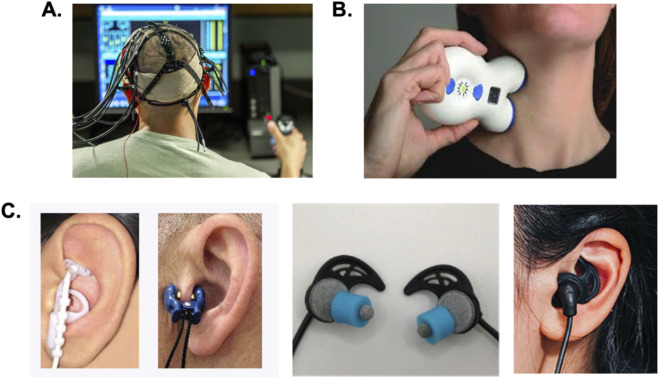
Wearable electrical neuromodulation approaches for augmenting human-machine interactions. Several forms of noninvasive neuromodulation are wearable and can be easily used before, during, or after virtual training activities on simulators or used during human-machine operations of drones or other robotic systems. **(A)** Transcranial electrical stimulation including transcranial direct and alternating current stimulation methods involve the placement of electrodes at different locations on the scalp to modulate cognitive, sensory, and motor networks. **(B)** Transcutaneous cervical vagus nerve stimulation involves placement of surface electrode contacts along the side of the neck to modulate activity of cervical branches of the vagus. **(C)** Several different approaches to transcutaneous auricular vagus nerve stimulation (taVNS) have been developed. As shown on the far-right some taVNS methods use small, steel ball electrodes to stimulate the external ear (Nemos device, Cerbomed GmbH). This approach can produce high, local current densities resulting in discomfort or electrical biting and stinging sensations. Other taVNS methods and devices utilize metal clip electrodes as shown in the middle-left image. These clips mechanically couple the skin to a metal electrode using an electrolyte solution or gel. This approach creates a distracting pinching sensation and can produce electrical biting or prickling sensations. Other methods were developed using conductive hydrogel earbud electrodes (BRAIN Buds, IST) to produce uniform current distributions and enhanced user comfort during taVNS.

Both tES and tVNS methods involve the transmission of weak (<4 mA) electrical currents across the skin. The general tES method embodies different approaches including transcranial direct current stimulation (tDCS) and transcranial alternating current stimulation (tACS) (Paulus, 2011). These methods different electrode sizes, shapes, densities, and polarities to deliver a DC or AC currents (1–20,000 Hz) across the skin of the scalp to bias the activity and plasticity of targeted cortical regions (Figure 3A). Another tES approach known as transcranial random noise stimulation (tRNS) is just like tACS, but delivers different pulsed or AC stimuli at random or pseudo-random frequencies (Paulus, 2011; Antal and Herrmann, 2016). Using transcutaneous electrical nerve stimulation approaches, tVNS may be achieved by targeting cervical branches of the vagus on the side of the neck in a method known as transcutaneous cervical vagus nerve stimulation (tcVNS; Figure 3B). Alternatively, transcutaneous auricular vagus nerve stimulation (taVNS) can be achieved by targeting auricular branches of the vagus nerve using pulsed stimuli located on the external ears (Figure 3C). All these methods have well understood mechanisms of action, offer comfortable and wearable human factors designs, can be safely used, and have been shown to improve various aspects of human behavior and cognition that can benefit remote operator performance as discussed below.

#### Transcranial electrical stimulation

The modern resurgence of tES was spawned by observations demonstrating that tDCS produces neurophysiological changes in cortical network excitability and brain plasticity (Nitsche and Paulus, 2000). Following this seminal observation, the field of noninvasive neuromodulation experienced explosive growth. A series of pioneering studies conducted by the US Air Force Research Laboratories provided the first evidence that cortical tES can be effective for enhancing remote operator cognition, training, performance (McKinley et al., 2011; McKinley et al., 2013; McIntire et al., 2014; Nelson et al., 2014). McKinley and colleagues (2013) initially demonstrated that 30-min tDCS (2 mA) can enhance remote operator training by producing a 25% improvement on visual search tasks compared to controls (McKinley et al., 2013). This work laid the foundation for follow-up investigations demonstrating tDCS applied to prefrontal cortex improves attention, reduces mental fatigue, and enhances multi-tasking during sustained, complex tasks that are ecologically valid and contextually relevant to remote drone and machine operations (McIntire et al., 2014; Nelson et al., 2014; Nelson et al., 2016).

Using EEG and fNIRS to record brain activity, it has been shown that tDCS delivered to the primary motor cortex (M1) increases neuronal excitability and enhances sensorimotor skill learning outcomes on a flight simulator (Choe et al., 2016). Targeting M1 using tDCS has been shown to enhance learning and performance on several other skilled motor tasks, such as sequential tapping and controlled force pinching tasks (Saucedo Marquez et al., 2013; Orban de Xivry and Shadmehr, 2014). Studies evaluating the influence of tES on motor vehicle operator performance have also shown improvements to executive function and cognitive performance. For example, a recent study demonstrated that 30-min tRNS delivered during VR truck driving simulation tasks significantly reduced mental fatigue compared to controls (Benelli et al., 2024). Another recent study showed tRNS can accelerate learning in VR environments (Neri et al., 2025). These data suggest that hybrid neurostimulation strategies incorporating VR or realistic simulations with tES may be particularly beneficial for remote operator training. Given that tDCS has been shown to protect against diminished executive vigilance decrement under high cognitive loads (Hemmerich et al., 2024), its application in long duration teleoperation training or missions may also significantly reduce operator fatigue and improve safety. Other research on driving simulators indicates that attentional control and reaction times can be significantly improved using tDCS, which further indicates tES methods holds promise for optimizing operator performance in dynamic, virtual, training or work environments (Facchin et al., 2023). Further research is required to identify the optimal parameters and brain targets for using tES methods before and during training or work procedures to improve remote operator attention and learning.

Evidence from investigations into the use of tDCS for treating various clinical conditions has evolved into insights that hold promise for enhancing general cognitive performance. Several studies have shown tDCS delivered to the prefrontal cortex can improve executive functions like working memory, impulse control, and cognitive flexibility, as well as time perception in children and adults with attention-deficit hyperactivity disorder (ADHD) (Nejati et al., 2020; Nejati et al., 2021; Nejati et al., 2024; Leffa et al., 2022). These observations combined with those described above showing tDCS improves driver skill training and performance, suggest tES may be useful for improving the safety of drivers with attention disorders like ADHD. In patient populations who exhibit problems with impulse control, such as those with gambling disorders, prefrontal tDCS has been shown to enhance cognitive control and decision making by reducing risk taking while improving cognitive flexibility (Soyata et al., 2019; Gilmore et al., 2018). In healthy adults tDCS can improve working memory, decision making, and impulse control (Ke et al., 2019; Ruf et al., 2017; Ouellet et al., 2015; Yang et al., 2017). Likewise, tACS delivered at theta and gamma frequencies can improve working memory and recall in healthy adults (Jaušovec et al., 2014; Hoy et al., 2015). Further, tACS delivered 4 days in a row can produce significant improvements in working and long-term memory that last up to 1 month in healthy aged adults (Grover et al., 2022). These cognitive enhancements would be beneficial to remote operators to improve skill learning and retention. The increasing prevalence of teleoperated machines and construction equipment justifies deeper explorations into how neuromodulation can improve attention and support adaptive decision-making by reducing risk taking, particularly in hazardous and high-risk environments (Lee et al., 2022). While the literature clearly supports these approaches, further investigations are required to understand how different stimulation intensities, frequencies, and locations differentially affect executive function during realistic remote operator training scenarios.

#### Transcutaneous vagus nerve stimulation

Using pulsed electrical currents to modulate vagus nerve fibers located in different locations along the side of the neck or external ear has gained attention for its safe ability to modulate autonomic nervous system activity, inflammation, neuroplasticity, attention, stress, learning, mood, and sleep (Tyler, 2017; Kim et al., 2022; Liu et al., 2020; Wang et al., 2021; Butt et al., 2020; Verma et al., 2021; Sant'Anna et al., 1992; Tan et al., 2023; Urbin et al., 2021; Bottari et al., 2024; Srinivasan et al., 2023; Ma et al., 2022; Phillips et al., 2021; Prescott and Liberles, 2022; Henry, 2002; Thayer and Sternberg, 2006). It is well established that noninvasive VNS works by modulating the activity of the locus coeruleus and ascending reticular activating system located in brainstem. This primary action alters the release of norepinephrine across large brain regions and organs in the body produces changes in activity, arousal, and plasticity (Urbin et al., 2021; Frangos et al., 2015; Sharon et al., 2021; Schuerman et al., 2021; Frangos and Komisaruk, 2017). Other neurotransmitters like acetylcholine (Martin et al., 2024; Pavlov and Tracey, 2005) and serotonin (Hulsey et al., 2019; Manta et al., 2009) have also been shown to regulate immune function and brain plasticity following transcutaneous VNS. Targeting cervical vagus nerve fibers using tcVNS is FDA cleared to treat headache, while targeting auricular vagus nerve fibers using taVNS is FDA cleared to treat opiate withdrawal. When proper precautions and methods are used, tcVNS and taVNS pose a low-risk or non-significant risk and have numerous other health and wellness applications. In fact, several tcVNS and taVNS devices are distributed over the counter as General Wellness devices when not used to treat or diagnose a disease or medical condition. Many studies in healthy volunteers demonstrate these noninvasive neuromodulation approaches can enhance human cognition and performance as further discussed below.

Several studies have shown that both tcVNS and taVNS can reduce the sympathetic nervous system activity, as well as the psychological and neurophysiological symptoms of stress (Trifilio et al., 2023; Bretherton et al., 2019; Clancy et al., 2014; Machetanz et al., 2021a; Machetanz et al., 2021b; Gurel et al., 2020; Moazzami et al., 2023). A recent randomized, sham-controlled study demonstrated that taVNS produced significant changes in bottom-up neurophysiological arousal leading to significantly improved impulse control during emotional tasks. It also been demonstrated that taVNS can improve cognitive control during multi-tasking to enhance performance (Sommer et al., 2023). The ability of taVNS to dampen stress responses is likely a contributing factor to the improved performance observed under high cognitive and emotional loads. In fact, taVNS has been shown to improve action control performance and response selection when task demands are high (Jongkees et al., 2018). The reduction of stress by taVNS also suggest it can be used following virtual or realistic training sessions to improve rest and recovery from mental strain or fatigue (Ferstl et al., 2022). Several studies have also shown that both taVNS and tcVNS can improve human learning and memory (Phillips et al., 2021; Kaan et al., 2021; Pandža et al., 2020; Jacobs et al., 2015; Olsen et al., 2023; Zhang et al., 2020; Cibulcova et al., 2024; Ridgewell et al., 2021; Miyatsu et al., 2024; Choudhary et al., 2023; Klaming et al., 2022).

**FIGURE 4:**
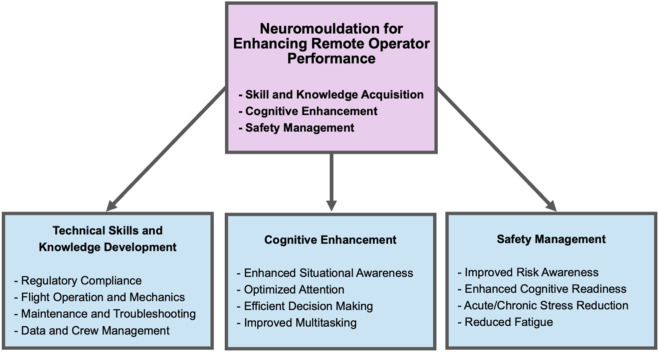
Noninvasive neuromodulation approaches for enhancing remote operator training and performance. The diagram illustrates how noninvasive neuromodulation methods may be used to enhance different aspects of human-machine operator performance by improving skill acquisition and retention, improving cognitive abilities, and improving operational safety.

In addition to direct effects on neuroplasticity, the influence of tVNS on learning and memory can be also attributed to its ability to modulate human cortical arousal and attention (Sharon et al., 2021; Trifilio et al., 2023; Miyatsu et al., 2024; Chen et al., 2023; Rufener et al., 2018; Giraudier et al., 2024; Tyler et al., 2019). Recent studies show that taVNS can significantly improve motor action planning, enhance motor sequence learning, and improve associated motor cortex efficiency (Chen et al., 2022; Chen et al., 2024). It has also been demonstrated that taVNS can improve human working memory (Sun et al., 2021) and cognitive flexibility (Borges et al., 2020). When subjects are sleep deprived, taVNS has been shown to reduce fatigue and improve working memory (Zhao et al., 2023), while tcVNS also reduces fatigue and improves multitasking performance (McIntire et al., 2021). Attention, working memory, and cognitive control networks are critical in decision making (Bechara et al., 1998; Curtis and Lee, 2010; McGuire and Botvinick, 2010; Cole and Schneider, 2007). Impaired working memory has been shown to underlie impulsivity in decision making (Hinson et al., 2003). These different approaches to enhancing cognition can prove beneficial to improving human-machine operator training and performance (Figure 4).

Impulsive decision-making by vehicle and remote operators represents a major risk since it can lead to dangerous situations or crashes injuring people and property. Interestingly, studies have demonstrated that taVNS can produce more efficient neural processing requiring fewer resources to achieve cognitive control (Pihlaja et al., 2020), as well as to improve cognitive control or adaptation in response to conflict (Fischer et al., 2018). As cited above, taVNS improves action control performance when task demands are high (Jongkees et al., 2018) and enhances cognitive control during multi-tasking (Sommer et al., 2023). These data indicate tVNS may improve cognitive control and flexibility, enabling vehicle and remote machine operators to switch between tasks and manage multiple streams of information more efficiently (Figure 4). Given that cognitive training has been shown to reduce motor vehicle collision involvement by up to 50% in older drivers (Ball et al., 2010), similar cognitive training approaches combined with tVNS to reinforce learning outcomes and executive control may help mitigate human error in remote industrial robotics and autonomous vehicle supervision. It has also been shown that taVNS boosts human drive to work for rewards suggesting it may be useful for improving the motivation of tele-operators, drivers, and remote pilots to engage in reward-based training (Neuser et al., 2020).

Studies have shown that tcVNS and taVNS provide roughly equal benefits. The choice to use one method or approach over another can be distilled down to human factors issues. One may consider whether they need hands-free capabilities for real-time modulation. One may also consider how one makes the operator feel from a sensory stimulation standpoint as this is becoming one of the key issues related to the use of tVNS for cognitive enhancement. For enhancing cognition and reducing stress it is critical that the user or patient has a comfortable experience where the stimulation is just noticeable or not noticeable from a sensory stimulation perspective. Otherwise the off target sensory effects that emerge as distracting and uncomfortable sensations from electrical stimulation can override intended tVNS outcomes (Miyatsu et al., 2024; Jigo et al., 2024). In other words, modulating vagus nerve activity using transcutaneous, pulsed electrical nerve stimulation methods can both activate and suppress sympathetic activity (stress) depending on many variables including the electrode interface, user sensation and comfort, stimulus frequency, pulse duration, ease of use, and others (Figures 3B,C). This has been observed in studies evaluating the influence of cognitive load and tVNS on pupillometry as a noradrenergic-related measure of neurophysiological arousal (Faller et al., 2019; Urbin et al., 2021; Phillips et al., 2021; Sharon et al., 2021; Pandža et al., 2020; Tyler et al., 2019; Ramakrishnan et al., 2021). Efforts aimed at improving human factors or neuroergonomics of tVNS can be combined with work to advance neurostimulation algorithms and parameters for continuing to enhance the electrical sensation experiences, ease of use, user comfort, and efficacy. This approach should prove valuable considering provocative demonstrations that high frequency (20,000 Hz), sub-perceptual taVNS produces significant changes in cerebellar activity (Chen et al., 2021), as well as changes in the functional connectivity of the prefrontal cortex, cingulate cortex, and insula.

Using vibrotactile and haptic stimulation of the external ear and vagus nerve has also shown potential for enhancing human-computer interactions. It has been argued the tactile sensitivity of the external ear has been overshadowed by its auditory functions and that haptic stimulation of the ear represents an opportunity for information transfer (Lee et al., 2019). This point reiterates the importance of ensuring sensations from stimulation are not distracting to the user (Tyler, 2025). Lee and colleagues (2019) demonstrated small ear worn haptic stimulation devices could encode environmentally relevant spatiotemporal information by stimulating six different locations on the external ear. In an adaptive embodiment, ear haptics were demonstrated as a human-computer interface to enhance the experience of virtual reality applications for deaf and hard-of-hearing (DHH) individuals (Mirzaei et al., 2020). Haptic stimulation of the ear can convey sound direction in relation to DHH users during a VR experience when a system was not universally designed and intended for hearing enabled persons using spatially encoded audio to simulate sound distance (Mirzaei et al., 2020). Combining different methods of vibrotactile and electrical stimulation may open new possibilities for modulation of human-machine cognition, such as to improve situational awareness and multimodal attention. Future human factors studies and engineering efforts should aim to identify and translate these methods to interoperate with traditional methods, such as cognitive training that have proven indispensable.

#### Transcranial focused ultrasound neuromodulation

Neuromodulation by transcranial focused ultrasound (tFUS) provides unrivaled spatial resolution and precision compared to other noninvasive modalities (Tyler et al., 2018). This method enables noninvasive, deep-brain stimulation in humans across many brain regions. Advancing tFUS or transcranial ultrasound stimulation (TUS) for human-machine interfaces has been a major interest since our pioneering studies demonstrating that low-intensity pulsed ultrasound can stimulate intact brain circuits (Tyler et al., 2008; Tufail et al., 2010). Ultrasonic neuromodulation and tFUS work when the sound waves of low-intensity, pulsed ultrasound mechanically modulate the electrical activity of brain circuits and nerves by altering the activity of pressure-sensitive ion channels, transporters, and neuronal membranes (Tyler et al., 2018; Tyler et al., 2008; Naor et al., 2016; Darmani et al., 2022). Depending on the frequency of ultrasound implemented, the spatial resolution of focused ultrasound for neuromodulation can achieve single cell resolutions *in vitro*, to a few microns in nerves, to a couple millimeters when delivered transcranial to deep-brain regions (Tyler et al., 2018; Naor et al., 2016). As discussed below, several lines of evidence demonstrate that tFUS can be useful for modulating human-machine cognition and interactions (Tyler, 2017; Tyler et al., 2018; Lee et al., 2024; Kim T. et al., 2021).

Modulation of human cortex to influence sensory processing and motor performance during training or teleoperation of machines may enhance human operator performance. The first functional evidence that 0.5 MHz tFUS can noninvasively modulate human brain activity recorded by EEG demonstrated that tactile discrimination abilities are enhanced following brief delivery of tFUS to the hand region of primary somatosensory cortex (Legon et al., 2014). In similar functional studies, tFUS delivered to the hand region of primary motor cortex has been shown to modulate brain circuit activity and enhance reaction times (Legon et al., 2018a; Fomenko et al., 2020). These approaches are particularly interesting options to optimize human interactions with joysticks, buttons, or hand controls by enhancing sensorimotor performance. A recent breakthrough study demonstrated the accurate, reliable, and individualized, deep-brain targeting of tFUS to the globus pallidus internus of the basal ganglia in Parkinson’s patients (Darmani et al., 2025). These observations combined with others demonstrating deep-brain modulation of motor and sensory thalamic nuclei (Legon et al., 2018b; Dallapiazza et al., 2018; Bancel et al., 2024) open the possibility of using tFUS to influence different nodes in human sensorimotor circuits for enhancing teleoperator performance. Another potential avenue is to modulate visual spatial processing by TUS. In support of this approach a recent study in healthy human volunteers showed tFUS delivered to visual cortex area 5 (V5) enhanced feature-based attention to motion by modulating activity in the dorsal visual processing pathway (Kosnoff et al., 2024). Interestingly, Kosnoff and colleagues (2024) also found this performance improvement led to reduced errors when subjects performed a spelling task using an EEG-based brain-computer interface (BCI) (Kosnoff et al., 2024). These findings raise the possibility of using tFUS to modulate human visual cortex and attention during robotic and semi-autonomous operation, as well as to improve BCI performance.

**FIGURE 5:**
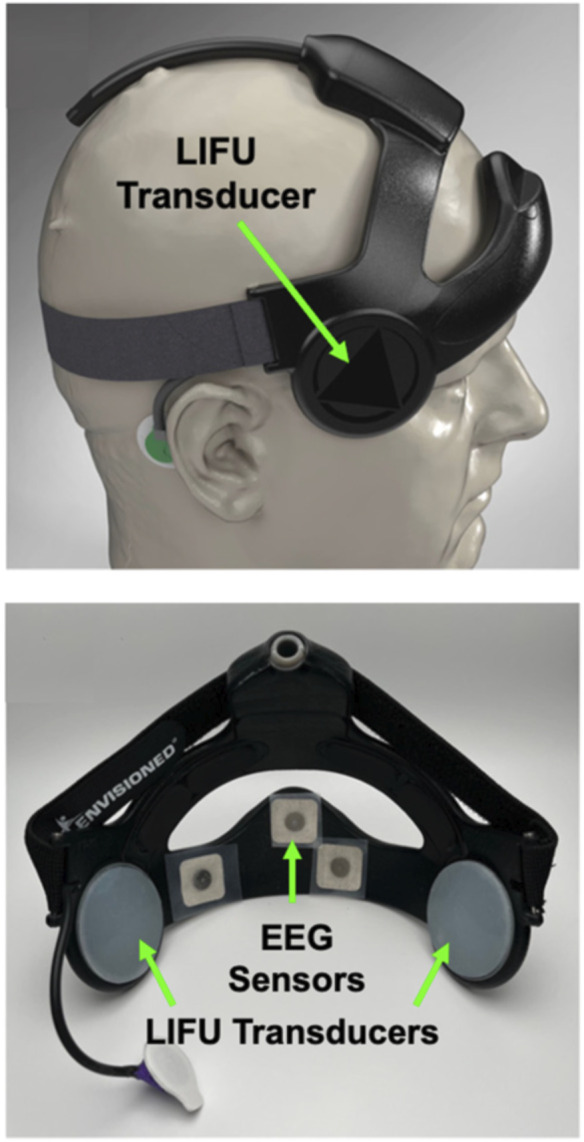
Wearable transcranial focused ultrasound neuromodulation devices. Recent breakthroughs in human factors, mechanical, materials, biomedical, and electrical engineering have enabled the development of wearable devices for neuromodulation by low-intensity transcranial focused ultrasound (LIFU). The device shown (Attune Neuroscience, Inc.) utilizes LIFU transducers to target different regions of the human brain and integrates electroencephalography (EEG) sensors for measuring brain wave activity patterns from the prefrontal cortex. This type of wearable device for neuromodulation by LIFU opens the possibility of developing closed-loop applications for human-in-the-loop control of robotic and semi-autonomous systems. Figure adapted from Reference (Meads et al., 2024).

Other targets and approaches for modulating the cognitive function of remote operators or pilots with tFUS should be considered. A recent study demonstrated that tFUS improved cognitive control when delivered to the right inferior frontal gyrus (rIFG), which helps to regulate cognitive aspects of behavioral response inhibition (Fine et al., 2023). Fine and colleagues (2023) showed that tFUS targeted to the rIFG significantly decreased P300 response latencies and enhanced response inhibition in healthy volunteers (Fine et al., 2023). Another recent study found faster reaction times to “go” signals in cognitive tasks following tFUS delivery to the right inferior frontal cortex (IFC) (Atkinson-Clement et al., 2024). Magnetic resonance imaging revealed the enhancement of reaction time was correlated with a decrease in functional connectivity between the IFC and post-central gyrus (Atkinson-Clement et al., 2024). There were also significant changes in the functional connectivity between IFC and the anterior cingulate cortex superior frontal cortex that evolved over 20–40 min following brief tFUS treatment (Atkinson-Clement et al., 2024). These observations collectively demonstrate that tFUS can be used to enhance various aspects of cognitive control networks in healthy humans. Until recently however, it has been difficult to explore how these approaches may be used in human-machine interfaces due to the power requirements and size of equipment needed to conduct tFUS and ultrasonic neuromodulation. Several engineering breakthroughs led to the development of miniaturized transducers and systems that could be used in various applications (Tyler et al., 2018). Remarkably, it has recently been demonstrated that wearable tFUS devices with integrated EEG sensors that are comfortable enough to wear during sleep (Meads et al., 2024) are useful for modulating deep-brain thalamic targets in humans (Figure 5) (Fan et al., 2024). Ongoing human factors, science, and engineering efforts should be aimed at advancing tFUS or TUS to enhance HMI’s and BCI’s in robotic and teleoperation.

### Brain-computer interfaces for augmenting teleoperation of robotic and semi-autonomous machines

Over the past several decades, the study, development, and testing of BCI’s spans many disciplines, philosophies, and approaches. Therefore, there has already been significant progress in advancing both noninvasive and invasive BCI hardware and software for various applications ranging from prosthetic limb control to providing sensory inputs to the brain to telerobotic operation to enhancing cognition (Hotson et al., 2016; Klaes et al., 2014; Golub et al., 2016; Tonin et al., 2020) (Duan et al., 2019; Arvaneh et al., 2019; Kryger et al., 2017). Here we restrict our discussion to noninvasive BCI approaches that can be readily scaled across healthy populations of teleoperators and remote pilots or machine operators. Integrating electroencephalographic EEG or fNIRS sensors into existing training and operations procedures can be readily accomplished to monitor cognitive load, stress, or attention. These approaches have been used, for example, to monitor the psychophysiological and cognitive states of drone pilots (Khan and Hong, 2017; Dell’Agnola et al., 2022; Dalilian and Nembhard, 2024). Some proposed taVNS-EEG closed-loop systems for modulating sleep, cognition, and attention can be designed to fit in the external ear resulting in a usability with a high degree of comfort posing minimal distractions to human operators (Tyler, 2025; Ruhnau and Zaehle, 2021). As discussed below, such designs and approaches will be advantageous to applications intended to enhance human teleoperator performance.

**FIGURE 6:**
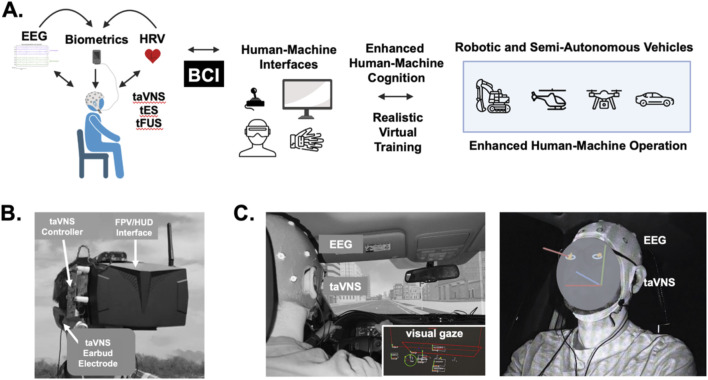
Brain-computer interface approaches for real-time optimization of remote pilot and driver performance. **(A)** Several types of biometric data including brain signals from electroencephalography (EEG), heart rate, and heart rate variability can be used to infer stress, attention, and fatigue in teleoperators and drivers. These biometric signals can used alone or in conjunction with signals and cues from human-machine interfaces and controllers as triggers or commands to elicit transcutaneous auricular vagus nerve stimulation (taVNS), transcranial electrical stimulation (tES), and transcranial focused ultrasound (tFUS) for enhancing the human operation of robotic machines and semi-autonomous vehicles. **(B)** The photographs illustrate a taVNS device integrated with a first-person view (FPV) display and headset receiving video feeds and telemetric data from a sUAS. This head-mounted taVNS approach is useful for delivering, real-time bilateral taVNS during FPV sUAS training and flight operations. **(C)** The photographs illustrate a subject wearing an EEG cap for monitoring attention and cognitive states while an eye tracking system is used to monitor visual attention and gaze during driving simulations. The subject is also wearing taVNS electrodes to responsively modulate attention and decision-making. Using EEG signals and data from the eye tracking system, taVNS can be triggered in a closed-loop manner that is responsive to an individual’s cognitive load, attention level, or stress to modulate operator neuroplasticity and cognition during training sessions in synthetic or virtual environments.

Several BCI studies have shown that brain signals recorded from EEG and fNIRS sensors can be used to directly control drone flight and behavior (Kim et al., 2014; Duan et al., 2019; Khan and Hong, 2017; Deng et al., 2023; Kim S. et al., 2021; Lee et al., 2021; Zafar et al., 2018). While these types of approaches are enticing to explore, we are limiting our discussion to potential BCI embodiments in which brain activity, cognitive networks, and behaviors of the human operators are modulated based on sensor information reporting engagement, work effort, attention, arousal/stress, and other environmental factors. We note this contrasts with BCIs where brain or physiological activity sensors and measures are used for machine control. Several physiological markers including EEG, eye movements, pupil dilation, and sudomotor activity have been to be reliable measures of attention and vigilance (Figure 6). While heart rate and respiration rate can reflect arousal and stress, EEG has also proven useful for monitoring other aspects of cognition including mental workload and fatigue, engagement, and abstraction (Chikhi et al., 2022; Dimitrova et al., 2021; Souza and Naves, 2021; Dmochowski et al., 2012; Antonenko et al., 2010; Kumar and Kumar, 2016).

Enhancing sustained attention or vigilance and reducing mental fatigue are feasible, early targets for demonstrating closed-loop, noninvasive neuromodulation BCIs intended to teleoperator performance and human-machine cognition. For example, triggering tFUS delivered to the rIFG in response to changes over time in the amplitudes or latencies of P300 potentials may be a relatively simple way to enhance teleoperator cognitive control during long periods (hours) of sustained operation. Other EEG signals, such as theta (4–8 Hz) and alpha (8–12 Hz) power from central-parietal brain regions during the pre-stimulus period, are reliable markers of psychomotor vigilance that have been used to predict response time (Chowdhury et al., 2020). Frontal theta power has been shown to be a reliable index of cognitive workload (Chikhi et al., 2022). A major limitation in the use of noninvasive BCI’s has been related to low ecological validity (lab-based vs. real-world studies) and low signal reliability. To address this several approaches are being pursued. For example, a recent study examining cognitive states in real-world environments showed increased frontal theta and decreased central delta (0–4 Hz) EEG brain wave amplitudes during periods of increased attention with the converse observed during distraction (Kaushik et al., 2022). The reliability of natural EEG brain signals can be greatly enhanced by inducing steady state evoked potentials (SSEPs). SSEPs are created by flickering stimuli on-and-off at a set frequency and can be generated with visual (Ding et al., 2006; Gulbinaite et al., 2019), auditory, and somatosensory stimuli (Muller-Putz et al., 2006). The increase in spectral power induced at the flicker frequency varies with attention and are often used in machine controlling BCI applications (Ding et al., 2006; Muller-Putz et al., 2006; Su et al., 2020; Giabbiconi et al., 2004). The spectral, power, and spatial characteristics of EEG and other biomarkers can be monitored to trigger tVNS and/or tFUS stimulation in a closed-loop system manner for augmenting human operator cognition and performance (Figure 6). Emerging data fusion efforts using multimodal sensing approaches combined with conventional machine learning methods will improve the reliability and scalability of closed-loop systems used for enhancing teleoperator cognition.

## Conclusion

Several neurotechnologies hold promise for improving human-machine cognition and performance during the training and operation of drones, robotic systems, and semi-autonomous vehicles. Evidence has shown that noninvasive neuromodulation methods like tES, tVNS, and tFUS can improve cognitive functions such as attention, learning, and memory, as well as sensorimotor abilities. These enhancements can produce unique benefits for remote pilots and robotic teleoperators, who face complex cognitive tasks in demanding operational environments. By incorporating these noninvasive neuromodulation approaches into training programs, we can create more resilient, focused, and efficient remote pilots and teleoperators, ultimately improving the safety and efficacy human-machine operations.

Looking forward, the applications of tES, tVNS, and tFUS as part of a broader neurotechnology strategy has the potential to transform HMI’s. Future research should continue to refine noninvasive neuromodulation targets and protocols while expanding its integration with other neurotechnologies, such as EEG BCIs, to develop closed-loop systems that provide real-time cognitive enhancement during teleoperation of robotic and semi-autonomous machines and drones. Additionally, as these technologies evolve, it will be essential to validate their long-term benefits in real-world scenarios, ensuring that they contribute not only to individual human operator performance but also to broader industrial safety and workforce readiness. The approaches discussed may be useful for solutions intended to improve HCIs in the larger robotics industry. For example, it should be explored whether open- and closed-loop neuromodulation approaches can be used to enhance other human-robot interactions, where we cooperate with machines using specific skills and knowledge to accomplish large or complex tasks in manufacturing, construction, medicine, shipping, transportation and delivery, city and vehicle maintenance, deep-sea and space exploration, and other areas. Through continued innovation and convergence research, different neurotechnologies and approaches discussed can help augment training and operational excellence in human-machine interactions.

## References

[B1] Adami P. Rodrigues P. B. Woods P. J. Becerik-Gerber B. Soibelman L. Copur-Gencturk Y. (2021). Effectiveness of VR-based training on improving construction workers’ knowledge, skills, and safety behavior in robotic teleoperation. Adv. Eng. Inf. 50, 101431. 10.1016/j.aei.2021.101431

[B2] Aggravi M. Pausé F. Giordano P. R. Pacchierotti C. (2018). Design and evaluation of a wearable haptic device for skin stretch, pressure, and vibrotactile stimuli. IEEE Robotics Automation Lett. 3, 2166–2173. 10.1109/lra.2018.2810887

[B3] Aguilar Reyes C. I. Wozniak D. Ham A. Zahabi M. (2023). Design and evaluation of an adaptive virtual reality training system. Virtual Real. 27, 2509–2528. 10.1007/s10055-023-00827-7

[B4] Antal A. Alekseichuk I. Bikson M. Brockmöller J. Brunoni A. R. Chen R. (2017). Low intensity transcranial electric stimulation: safety, ethical, legal regulatory and application guidelines. Clin. Neurophysiol. 128, 1774–1809. 10.1016/j.clinph.2017.06.001 28709880 PMC5985830

[B5] Antal A. Herrmann C. S. (2016). Transcranial alternating current and random noise stimulation: possible mechanisms. Neural Plast. 2016, 1–12. 10.1155/2016/3616807 PMC4868897 27242932

[B6] Antal A. Luber B. Brem A.-K. Bikson M. Brunoni A. R. Cohen Kadosh R. (2022). Non-invasive brain stimulation and neuroenhancement. Clin. Neurophysiol. Pract. 7, 146–165. 10.1016/j.cnp.2022.05.002 35734582 PMC9207555

[B7] Antonenko P. Paas F. Grabner R. van Gog T. (2010). Using electroencephalography to measure cognitive load. Educ. Psychol. Rev. 22, 425–438. 10.1007/s10648-010-9130-y

[B8] Arvaneh M. Robertson I. H. Ward T. E. (2019). A P300-based brain-computer interface for improving attention. Front. Hum. Neurosci. 12, 524. 10.3389/fnhum.2018.00524 30662400 PMC6328468

[B9] Atkinson-Clement C. Alkhawashki M. Gatica M. Ross J. Kaiser M. (2024). Dynamic changes in human brain connectivity following ultrasound neuromodulation. Sci. Rep. 14, 30025. 10.1038/s41598-024-81102-w 39627315 PMC11614892

[B10] Ball K. Edwards J. D. Ross L. A. McGwin J. (2010). Cognitive training decreases motor vehicle collision involvement of older drivers. J. Am. Geriatrics Soc. 58, 2107–2113. 10.1111/j.1532-5415.2010.03138.x PMC3057872 21054291

[B11] Bancel T. Béranger B. Daniel M. Didier M. Santin M. Rachmilevitch I. (2024). Sustained reduction of essential tremor with low-power non-thermal transcranial focused ultrasound stimulations in humans. Brain Stimul. 17, 636–647. 10.1016/j.brs.2024.05.003 38734066

[B12] Bechara A. Damasio H. Tranel D. Anderson S. W. (1998). Dissociation of working memory from decision making within the human prefrontal cortex. J. Neurosci. 18, 428–437. 10.1523/jneurosci.18-01-00428.1998 9412519 PMC6793407

[B13] Benelli A. Memoli C. Neri F. Romanella S. M. Cinti A. Giannotta A. (2024). Reduction of cognitive fatigue and improved performance at a VR-based driving simulator using tRNS. iScience 27, 110536. 10.1016/j.isci.2024.110536 39314236 PMC11418143

[B14] Bhattacharya A. Mrudula K. Sreepada S. S. Sathyaprabha T. N. Pal P. K. Chen R. (2022). An overview of noninvasive brain stimulation: basic principles and clinical applications. Can. J. Neurol. Sci. 49, 479–492. 10.1017/cjn.2021.158 34238393

[B15] Borges U. Knops L. Laborde S. Klatt S. Raab M. (2020). Transcutaneous vagus nerve stimulation may enhance only specific aspects of the core executive functions. A randomized crossover trial. Front. Neurosci. 14, 523. 10.3389/fnins.2020.00523 32523510 PMC7262369

[B16] Bottari S. A. Lamb D. G. Porges E. C. Murphy A. J. Tran A. B. Ferri R. (2024). Preliminary evidence of transcutaneous vagus nerve stimulation effects on sleep in veterans with post-traumatic stress disorder. J. Sleep Res. 33, e13891. 10.1111/jsr.13891 37039398

[B17] Bretherton B. Atkinson L. Murray A. Clancy J. Deuchars S. Deuchars J. (2019). Effects of transcutaneous vagus nerve stimulation in individuals aged 55 years or above: potential benefits of daily stimulation. Aging 11, 4836–4857. 10.18632/aging.102074 31358702 PMC6682519

[B18] Burk E. Han H.-S. Smidt M. Fox B. (2023). Effectiveness of simulator training compared to machine training for equipment operators in the logging industry. Int. J. For. Eng. 34, 373–384. 10.1080/14942119.2023.2194751

[B19] Butt M. F. Albusoda A. Farmer A. D. Aziz Q. (2020). The anatomical basis for transcutaneous auricular vagus nerve stimulation. J. Anat. 236, 588–611. 10.1111/joa.13122 31742681 PMC7083568

[B20] Chen C. Mao Y. Falahpour M. MacNiven K. H. Heit G. Sharma V. (2021). Effects of sub-threshold transcutaneous auricular vagus nerve stimulation on cerebral blood flow. Sci. Rep. 11, 24018. 10.1038/s41598-021-03401-w 34912017 PMC8674256

[B21] Chen L. Tang C. Wang Z. Zhang L. Gu B. Liu X. (2024). Enhancing motor sequence learning via transcutaneous auricular vagus nerve stimulation (taVNS): an EEG study. IEEE J. Biomed. Health Inf. 28, 1285–1296. 10.1109/jbhi.2023.3344176 38109248

[B22] Chen L. Zhang J. Wang Z. Zhang X. Zhang L. Xu M. (2022). Effects of transcutaneous vagus nerve stimulation (tVNS) on action planning: a behavioural and EEG study. IEEE Trans. Neural Syst. Rehabilitation Eng. 30, 1675–1683. 10.1109/tnsre.2021.3131497 34847035

[B23] Chen Y. Lu X. Hu L. (2023). Transcutaneous auricular vagus nerve stimulation facilitates cortical arousal and alertness. Int. J. Environ. Res. Public Health 20, 1402. 10.3390/ijerph20021402 36674156 PMC9859411

[B24] Chikhi S. Matton N. Blanchet S. (2022). EEG power spectral measures of cognitive workload: a meta-analysis. Psychophysiology 59, e14009. 10.1111/psyp.14009 35128686

[B25] Chirgwin P. (2021). Skills development and training of future workers in mining automation control rooms. Comput. Hum. Behav. Rep. 4, 100115. 10.1016/j.chbr.2021.100115

[B26] Choe J. Coffman B. A. Bergstedt D. T. Ziegler M. D. Phillips M. E. (2016). Transcranial direct current stimulation modulates neuronal activity and learning in pilot training. Front. Hum. Neurosci. 10, 34. 10.3389/fnhum.2016.00034 26903841 PMC4746294

[B27] Choudhary T. Elliott M. Euliano N. R. Gurel N. Z. Rivas A. G. Wittbrodt M. T. (2023). Effect of transcutaneous cervical vagus nerve stimulation on declarative and working memory in patients with Posttraumatic Stress Disorder (PTSD): a pilot study. J. Affect Disord. 339, 418–425. 10.1016/j.jad.2023.07.025 37442455 PMC11940650

[B28] Chowdhury M. S. Dutta A. Robison M. K. Blais C. Brewer G. A. Bliss D. W. (2020). Deep neural network for visual stimulus-based reaction time estimation using the periodogram of single-trial EEG. Sensors 20, 6090. 10.3390/s20216090 33120869 PMC7662233

[B29] Cibulcova V. Koenig J. Jackowska M. Jandackova V. K. (2024). Influence of a 2-week transcutaneous auricular vagus nerve stimulation on memory: findings from a randomized placebo controlled trial in non-clinical adults. Clin. Aut. Res. 34, 447–462. 10.1007/s10286-024-01053-0 PMC11732881 39039354

[B30] Cinel C. Valeriani D. Poli R. (2019). Neurotechnologies for human cognitive augmentation: current state of the art and future prospects. Front. Hum. Neurosci. 13, 13. 10.3389/fnhum.2019.00013 30766483 PMC6365771

[B31] Clancy J. A. Mary D. A. Witte K. K. Greenwood J. P. Deuchars S. A. Deuchars J. (2014). Non-invasive vagus nerve stimulation in healthy humans reduces sympathetic nerve activity. Brain Stimul. 7, 871–877. 10.1016/j.brs.2014.07.031 25164906

[B32] Cole M. W. Schneider W. (2007). The cognitive control network: integrated cortical regions with dissociable functions. NeuroImage 37, 343–360. 10.1016/j.neuroimage.2007.03.071 17553704

[B33] Colzato L. S. Nitsche M. A. Kibele A. (2017). Noninvasive brain stimulation and neural entrainment enhance athletic performance—a review. J. Cognitive Enhanc. 1, 73–79. 10.1007/s41465-016-0003-2

[B34] Cross E. S. Ramsey R. (2021). Mind meets machine: towards a cognitive science of human–machine interactions. Trends Cognitive Sci. 25, 200–212. 10.1016/j.tics.2020.11.009 33384213

[B35] Curtis C. E. Lee D. (2010). Beyond working memory: the role of persistent activity in decision making. Trends Cognitive Sci. 14, 216–222. 10.1016/j.tics.2010.03.006 PMC2883296 20381406

[B36] D’Abbraccio J. Massari L. Prasanna S. Baldini L. Sorgini F. Airò Farulla G. (2019). Haptic glove and platform with gestural control for neuromorphic tactile sensory feedback in medical telepresence. Sensors 19, 641. 10.3390/s19030641 30717482 PMC6386988

[B37] Dalilian F. Nembhard D. (2024). Cognitive and behavioral markers for human detection error in AI-assisted bridge inspection. Appl. Ergon. 121, 104346. 10.1016/j.apergo.2024.104346 39018705

[B38] Dallapiazza R. F. Timbie K. F. Holmberg S. Gatesman J. Lopes M. B. Price R. J. (2018). Noninvasive neuromodulation and thalamic mapping with low-intensity focused ultrasound. J. Neurosurg. JNS 128, 875–884. 10.3171/2016.11.jns16976 PMC7032074 28430035

[B39] Darmani G. Bergmann T. O. Butts Pauly K. Caskey C. F. de Lecea L. Fomenko A. (2022). Non-invasive transcranial ultrasound stimulation for neuromodulation. Clin. Neurophysiol. 135, 51–73. 10.1016/j.clinph.2021.12.010 35033772

[B40] Darmani G. Ramezanpour H. Sarica C. Annirood R. Grippe T. Nankoo J.-F. (2025). Individualized non-invasive deep brain stimulation of the basal ganglia using transcranial ultrasound stimulation. Nat. Commun. 16, 2693. 10.1038/s41467-025-57883-7 40108143 PMC11923056

[B41] Dell’Agnola F. Jao P. K. Arza A. Chavarriaga R. Millán J. d.R. Floreano D. (2022). Machine-learning based monitoring of cognitive workload in rescue missions with drones. IEEE J. Biomed. Health Inf. 26, 4751–4762. 10.1109/jbhi.2022.3186625 35759604

[B42] Deng T. Huo Z. Zhang L. Dong Z. Niu L. Kang X. (2023). A VR-based BCI interactive system for UAV swarm control. Biomed. Signal Process. Control 85, 104944. 10.1016/j.bspc.2023.104944

[B43] Dimitrova M. Wagatsuma H. Krastev A. Vrochidou E. Nunez-Gonzalez J. D. (2021). A review of possible EEG markers of abstraction, attentiveness, and memorisation in cyber-physical systems for special education. Front. Robotics AI 8, 715962. 10.3389/frobt.2021.715962 PMC8439420 34532347

[B44] Ding J. Sperling G. Srinivasan R. (2006). Attentional modulation of SSVEP power depends on the network tagged by the flicker frequency. Cereb. Cortex 16, 1016–1029. 10.1093/cercor/bhj044 16221931 PMC1880883

[B45] Dmochowski J. P. Sajda P. Dias J. Parra L. C. (2012). Correlated components of ongoing EEG point to emotionally laden attention – a possible marker of engagement? Front. Hum. Neurosci. 6, 112. 10.3389/fnhum.2012.00112 22623915 PMC3353265

[B46] Duan X. Xie S. Xie X. Meng Y. Xu Z. (2019). Quadcopter flight control using a non-invasive multi-modal brain computer interface. Front. Neurorobotics 13, 23. 10.3389/fnbot.2019.00023 PMC6554428 31214009

[B47] Edmondson R. Light K. Bodenhamer A. Bosscher P. Wilkinson L. (2012). Enhanced operator perception through 3D vision and haptic feedback, 8387, 838711, 10.1117/12.919306

[B48] Facchin A. La Rocca S. Vacchi L. Daini R. Gobbo M. Fontana S. (2023). Effects of conventional and high-definition transcranial direct current stimulation (tDCS) on driving abilities: a tDCS-driving simulator study. J. Environ. Psychol. 90, 102111. 10.1016/j.jenvp.2023.102111

[B49] Faller J. Cummings J. Saproo S. Sajda P. (2019). Regulation of arousal via online neurofeedback improves human performance in a demanding sensory-motor task. Proc. Natl. Acad. Sci. 116, 6482–6490. 10.1073/pnas.1817207116 30862731 PMC6442591

[B50] Fan J. M. Woodworth K. Murphy K. R. Hinkley L. Cohen J. L. Yoshimura J. (2024). Thalamic transcranial ultrasound stimulation in treatment resistant depression. Brain Stimul. Basic, Transl. Clin. Res. Neuromodulation 17, 1001–1004. 10.1016/j.brs.2024.08.006 PMC11531731 39173737

[B51] Feltman K. A. Kelley A. M. (2024). Transcranial direct current stimulation and aviator performance during simulated flight. Aerosp. Med. Hum. Perform. 95, 5–15. 10.3357/amhp.6243.2024 38158568

[B52] Ferstl M. Teckentrup V. Lin W. M. Kräutlein F. Kühnel A. Klaus J. (2022). Non-invasive vagus nerve stimulation boosts mood recovery after effort exertion. Psychol. Med. 52, 3029–3039. 10.1017/s0033291720005073 33586647 PMC9693679

[B53] Fine J. M. Mysore A. S. Fini M. E. Tyler W. J. Santello M. (2023). Transcranial focused ultrasound to human rIFG improves response inhibition through modulation of the P300 onset latency. Elife 12, e86190. 10.7554/elife.86190 38117053 PMC10796145

[B54] Fischer R. Ventura-Bort C. Hamm A. Weymar M. (2018). Transcutaneous vagus nerve stimulation (tVNS) enhances conflict-triggered adjustment of cognitive control. Cognitive, Affect. and Behav. Neurosci. 18, 680–693. 10.3758/s13415-018-0596-2 29693214

[B55] Floreano D. Wood R. J. (2015). Science, technology and the future of small autonomous drones. Nature 521, 460–466. 10.1038/nature14542 26017445

[B56] Fomenko A. Chen K.-H. S. Nankoo J.-F. Saravanamuttu J. Wang Y. El-Baba M. (2020). Systematic examination of low-intensity ultrasound parameters on human motor cortex excitability and behavior. eLife 9, e54497. 10.7554/elife.54497 33236981 PMC7728443

[B57] Frangos E. Ellrich J. Komisaruk B. R. (2015). Non-invasive access to the vagus nerve central projections via electrical stimulation of the external ear: fMRI evidence in humans. Brain Stimul. 8, 624–636. 10.1016/j.brs.2014.11.018 25573069 PMC4458242

[B58] Frangos E. Komisaruk B. R. (2017). Access to vagal projections via cutaneous electrical stimulation of the neck: fMRI evidence in healthy humans. Brain Stimul. 10, 19–27. 10.1016/j.brs.2016.10.008 28104084

[B59] Gani A. Pickering O. Ellis C. Sabri O. Pucher P. (2022). Impact of haptic feedback on surgical training outcomes: a Randomised Controlled Trial of haptic versus non-haptic immersive virtual reality training. Ann. Med. Surg. 83, 104734. 10.1016/j.amsu.2022.104734 PMC9661648 36389184

[B60] Giabbiconi C. M. Dancer C. Zopf R. Gruber T. Müller M. M. (2004). Selective spatial attention to left or right hand flutter sensation modulates the steady-state somatosensory evoked potential. Cognitive Brain Res. 20, 58–66. 10.1016/s0926-6410(04)00036-9 15130590

[B61] Gilmore C. S. Dickmann P. J. Nelson B. G. Lamberty G. J. Lim K. O. (2018). Transcranial Direct Current Stimulation (tDCS) paired with a decision-making task reduces risk-taking in a clinically impulsive sample. Brain Stimul. 11, 302–309. 10.1016/j.brs.2017.11.011 29174303

[B62] Giraudier M. Ventura-Bort C. Weymar M. (2024). Effects of transcutaneous auricular vagus nerve stimulation on the P300: do stimulation duration and stimulation type matter? Brain Sci. 14, 690. 10.3390/brainsci14070690 39061430 PMC11274684

[B63] Golub M. D. Chase S. M. Batista A. P. Yu B. M. (2016). Brain–computer interfaces for dissecting cognitive processes underlying sensorimotor control. Curr. Opin. Neurobiol. 37, 53–58. 10.1016/j.conb.2015.12.005 26796293 PMC4860084

[B64] Goode N. Salmon P. M. Lenné M. G. (2013). Simulation-based driver and vehicle crew training: applications, efficacy and future directions. Appl. Ergon. 44, 435–444. 10.1016/j.apergo.2012.10.007 23122978

[B65] Grasso G. Rosset S. Shea H. (2023). Fully 3D-printed, stretchable, and conformable haptic interfaces. Adv. Funct. Mater. 33, 2213821. 10.1002/adfm.202213821

[B66] Grover S. Fayzullina R. Bullard B. M. Levina V. Reinhart R. M. G. (2023). A meta-analysis suggests that tACS improves cognition in healthy, aging, and psychiatric populations. Sci. Transl. Med. 15, eabo2044. 10.1126/scitranslmed.abo2044 37224229 PMC10860714

[B67] Grover S. Wen W. Viswanathan V. Gill C. T. Reinhart R. M. G. (2022). Long-lasting, dissociable improvements in working memory and long-term memory in older adults with repetitive neuromodulation. Nat. Neurosci. 25, 1237–1246. 10.1038/s41593-022-01132-3 35995877 PMC10068908

[B68] Gulbinaite R. Roozendaal D. H. M. VanRullen R. (2019). Attention differentially modulates the amplitude of resonance frequencies in the visual cortex. NeuroImage 203, 116146. 10.1016/j.neuroimage.2019.116146 31493535

[B69] Gurel N. Z. Wittbrodt M. T. Jung H. Shandhi M. M. H. Driggers E. G. Ladd S. L. (2020). Transcutaneous cervical vagal nerve stimulation reduces sympathetic responses to stress in posttraumatic stress disorder: a double-blind, randomized, sham controlled trial. Neurobiol. Stress 13, 100264. 10.1016/j.ynstr.2020.100264 33344717 PMC7739181

[B70] Han H.-S. Smidt M. Fox B. Burk E. (2023). Incorporating simulators into a training curriculum for forestry equipment operators: a literature review. Croat. J. For. Eng. 45, 199–215. 10.5552/crojfe.2024.2142

[B71] Haruna M. Kawaguchi N. Ogino M. Koike-Akino T. (2021). Comparison of three feedback modalities for haptics sensation in remote machine manipulation. IEEE Robotics Automation Lett. 6, 5040–5047. 10.1109/lra.2021.3070301

[B72] Hemmerich K. Lupiáñez J. Martín-Arévalo E. (2024). HD-tDCS mitigates the executive vigilance decrement only under high cognitive demands. Sci. Rep. 14, 7865. 10.1038/s41598-024-57917-y 38570619 PMC10991279

[B73] Henry T. R. (2002). Therapeutic mechanisms of vagus nerve stimulation. Neurology 59, S3–S14. 10.1212/wnl.59.6_suppl_4.s3 12270962

[B74] Herrmann C. S. Rach S. Neuling T. Strüber D. (2013). Transcranial alternating current stimulation: a review of the underlying mechanisms and modulation of cognitive processes. Front. Hum. Neurosci. 7, 279. 10.3389/fnhum.2013.00279 23785325 PMC3682121

[B75] Hiltunen M. Heikkilä R. Niskanen I. Immonen M. (2023). Open InfraBIM for remote and autonomous excavation. Automation Constr. 156, 105148. 10.1016/j.autcon.2023.105148

[B76] Hinson J. M. Jameson T. L. Whitney P. (2003). Impulsive decision making and working memory. J. Exp. Psychol. Learn. Mem. Cognition 29, 298–306. 10.1037/0278-7393.29.2.298 12696817

[B77] Hotson G. McMullen D. P. Fifer M. S. Johannes M. S. Katyal K. D. Para M. P. (2016). Individual finger control of a modular prosthetic limb using high-density electrocorticography in a human subject. J. Neural Eng. 13, 026017. 10.1088/1741-2560/13/2/026017 26863276 PMC4875758

[B78] Hoy K. E. Bailey N. Arnold S. Windsor K. John J. Daskalakis Z. J. (2015). The effect of γ-tACS on working memory performance in healthy controls. Brain Cognition 101, 51–56. 10.1016/j.bandc.2015.11.002 26580743

[B79] Hulsey D. R. Shedd C. M. Sarker S. F. Kilgard M. P. Hays S. A. (2019). Norepinephrine and serotonin are required for vagus nerve stimulation directed cortical plasticity. Exp. Neurol. 320, 112975. 10.1016/j.expneurol.2019.112975 31181199 PMC6708444

[B80] Jacobs H. I. L. Riphagen J. M. Razat C. M. Wiese S. Sack A. T. (2015). Transcutaneous vagus nerve stimulation boosts associative memory in older individuals. Neurobiol. Aging 36, 1860–1867. 10.1016/j.neurobiolaging.2015.02.023 25805212

[B81] Jaušovec N. Jaušovec K. Pahor A. (2014). The influence of theta transcranial alternating current stimulation (tACS) on working memory storage and processing functions. Acta Psychol. 146, 1–6. 10.1016/j.actpsy.2013.11.011 24361739

[B82] Jigo M. Carmel J. B. Wang Q. Rodenkirch C. (2024). Transcutaneous cervical vagus nerve stimulation improves sensory performance in humans: a randomized controlled crossover pilot study. Sci. Rep. 14, 3975. 10.1038/s41598-024-54026-8 38368486 PMC10874458

[B83] Jongkees B. J. Immink M. A. Finisguerra A. Colzato L. S. (2018). Transcutaneous vagus nerve stimulation (tVNS) enhances response selection during sequential action. Front. Psychol. 9, 1159. 10.3389/fpsyg.2018.01159 30034357 PMC6043681

[B84] Kaan E. De Aguiar I. Clarke C. Lamb D. G. Williamson J. B. Porges E. C. (2021). A transcutaneous vagus nerve stimulation study on verbal order memory. J. Neurolinguistics 59, 100990. 10.1016/j.jneuroling.2021.100990

[B85] Kaushik P. Moye A. Vugt M. v. Roy P. P. (2022). Decoding the cognitive states of attention and distraction in a real-life setting using EEG. Sci. Rep. 12, 20649. 10.1038/s41598-022-24417-w 36450871 PMC9712397

[B86] Ke Y. Wang N. Du J. Kong L. Liu S. Xu M. (2019). The effects of transcranial direct current stimulation (tDCS) on working memory training in healthy young adults. Front. Hum. Neurosci. 13, 19. 10.3389/fnhum.2019.00019 30774590 PMC6367257

[B87] Khan M. J. Hong K.-S. (2017). Hybrid EEG–fNIRS-based eight-command decoding for BCI: application to quadcopter control. Front. Neurorobotics 11, 6. 10.3389/fnbot.2017.00006 PMC5314821 28261084

[B88] Kim A. Y. Marduy A. de Melo P. S. Gianlorenco A. C. Kim C. K. Choi H. (2022). Safety of transcutaneous auricular vagus nerve stimulation (taVNS): a systematic review and meta-analysis. Sci. Rep. 12, 22055. 10.1038/s41598-022-25864-1 36543841 PMC9772204

[B89] Kim B. H. Kim M. Jo S. (2014). Quadcopter flight control using a low-cost hybrid interface with EEG-based classification and eye tracking. Comput. Biol. Med. 51, 82–92. 10.1016/j.compbiomed.2014.04.020 24880998

[B90] Kim S. Lee S. Kang H. Kim S. Ahn M. (2021b). P300 brain–computer interface-based drone control in virtual and augmented reality. Sensors 21, 5765. 10.3390/s21175765 34502655 PMC8434009

[B91] Kim T. Park C. Chhatbar P. Y. Feld J. Mac Grory B. Nam C. S. (2021a). Effect of low intensity transcranial ultrasound stimulation on neuromodulation in animals and humans: an updated systematic review. Front. Neurosci. 15, 620863. 10.3389/fnins.2021.620863 33935626 PMC8079725

[B92] Klaes C. Shi Y. Kellis S. Minxha J. Revechkis B. Andersen R. A. (2014). A cognitive neuroprosthetic that uses cortical stimulation for somatosensory feedback. J. Neural Eng. 11, 056024. 10.1088/1741-2560/11/5/056024 25242377 PMC4410973

[B93] Klaming R. Simmons A. N. Spadoni A. D. Lerman I. (2022). Effects of noninvasive cervical vagal nerve stimulation on cognitive performance but not brain activation in healthy adults. Neuromodulation Technol. A. T. Neural Interface 25, 424–432. 10.1111/ner.13313 PMC8144242 35396072

[B94] Kosnoff J. Yu K. Liu C. He B. (2024). Transcranial focused ultrasound to V5 enhances human visual motion brain-computer interface by modulating feature-based attention. Nat. Commun. 15, 4382. 10.1038/s41467-024-48576-8 38862476 PMC11167030

[B95] Kryger M. Wester B. Pohlmeyer E. A. Rich M. John B. Beaty J. (2017). Flight simulation using a Brain-Computer Interface: a pilot, pilot study. Exp. Neurol. 287, 473–478. 10.1016/j.expneurol.2016.05.013 27196543

[B96] Kumar N. Kumar J. (2016). Measurement of cognitive load in HCI systems using EEG power spectrum: an experimental study. Procedia Comput. Sci. 84, 70–78. 10.1016/j.procs.2016.04.068

[B97] Lee D. H. Jeong J. H. Ahn H. J. Lee S. W. (2021). “Design of an EEG-based drone swarm control system using endogenous BCI paradigms,” in 2021 9th international winter conference on brain-computer interface (BCI), 1–5.

[B98] Lee J. S. Ham Y. Park H. Kim J. (2022). Challenges, tasks, and opportunities in teleoperation of excavator toward human-in-the-loop construction automation. Automation Constr. 135, 104119. 10.1016/j.autcon.2021.104119

[B99] Lee K. Park T. Y. Lee W. Kim H. (2024). A review of functional neuromodulation in humans using low-intensity transcranial focused ultrasound. Biomed. Eng. Lett. 14, 407–438. 10.1007/s13534-024-00369-0 38645585 PMC11026350

[B100] Lee M. Je S. Lee W. Ashbrook D. Bianchi A. (2019). ActivEarring: spatiotemporal haptic cues on the ears. IEEE Trans. Haptics 12, 554–562. 10.1109/toh.2019.2925799 31265405

[B101] Leffa D. T. Grevet E. H. Bau C. H. D. Schneider M. Ferrazza C. P. da Silva R. F. (2022). Transcranial direct current stimulation vs sham for the treatment of inattention in adults with attention-deficit/hyperactivity disorder: the TUNED randomized clinical trial. JAMA Psychiatry 79, 847–856. 10.1001/jamapsychiatry.2022.2055 35921102 PMC9350846

[B102] Legon W. Ai L. Bansal P. Mueller J. K. (2018b). Neuromodulation with single-element transcranial focused ultrasound in human thalamus. Hum. Brain Mapp. 39, 1995–2006. 10.1002/hbm.23981 29380485 PMC6866487

[B103] Legon W. Bansal P. Tyshynsky R. Ai L. Mueller J. K. (2018a). Transcranial focused ultrasound neuromodulation of the human primary motor cortex. Sci. Rep. 8, 10007. 10.1038/s41598-018-28320-1 29968768 PMC6030101

[B104] Legon W. Sato T. F. Opitz A. Mueller J. Barbour A. Williams A. (2014). Transcranial focused ultrasound modulates the activity of primary somatosensory cortex in humans. Nat. Neurosci. 17, 322–329. 10.1038/nn.3620 24413698

[B105] Liu A. M. Oman C. M. Galvan R. Natapoff A. (2013). Predicting space telerobotic operator training performance from human spatial ability assessment. Acta Astronaut. 92, 38–47. 10.1016/j.actaastro.2012.04.004

[B106] Liu C.-H. Yang M.-H. Zhang G.-Z. Wang X.-X. Li B. Li M. (2020). Neural networks and the anti-inflammatory effect of transcutaneous auricular vagus nerve stimulation in depression. J. Neuroinflammation 17, 54. 10.1186/s12974-020-01732-5 32050990 PMC7017619

[B107] Ljungblad S. Man Y. Baytaş M. A. Gamboa M. Obaid M. Fjeld M. (2021). “What matters in professional drone pilots’ practice? An interview study to understand the complexity of their work and inform human-drone interaction research,” in Proceedings of the 2021 CHI conference on human factors in computing systems (New York, NY: Association for Computing Machinery), 159.

[B108] Ma Y. Wang Z. He J. Sun J. Guo C. Du Z. (2022). Transcutaneous auricular vagus nerve immediate stimulation treatment for treatment-resistant depression: a functional magnetic resonance imaging study. Front. Neurology 13, 931838. 10.3389/fneur.2022.931838 PMC9477011 36119681

[B109] Macchini M. Havy T. Weber A. Schiano F. Floreano D. (2020). Hand-worn haptic interface for drone teleoperation. IEEE International Conference on Robotics and Automation ICRA, 10212–10218.

[B110] Machetanz K. Berelidze L. Guggenberger R. Gharabaghi A. (2021a). Transcutaneous auricular vagus nerve stimulation and heart rate variability: analysis of parameters and targets. Aut. Neurosci. 236, 102894. 10.1016/j.autneu.2021.102894 34662844

[B111] Machetanz K. Berelidze L. Guggenberger R. Gharabaghi A. (2021b). Brain–heart interaction during transcutaneous auricular vagus nerve stimulation. Front. Neurosci. 15, 632697. 10.3389/fnins.2021.632697 33790736 PMC8005577

[B112] Mallela A. N. Beiriger J. Gersey Z. C. Shariff R. K. Gonzalez S. M. Agarwal N. (2022). Targeting the future: developing a training curriculum for robotic assisted Neurosurgery. World Neurosurg. 167, e770–e777. 10.1016/j.wneu.2022.08.076 36030012

[B113] Manta S. Dong J. Debonnel G. Blier P. (2009). Optimization of vagus nerve stimulation parameters using the firing activity of serotonin neurons in the rat dorsal raphe. Eur. Neuropsychopharmacol. 19, 250–255. 10.1016/j.euroneuro.2008.12.001 19150228

[B114] Martin K. A. Papadoyannis E. S. Schiavo J. K. Fadaei S. S. Issa H. A. Song S. C. (2024). Vagus nerve stimulation recruits the central cholinergic system to enhance perceptual learning. Nat. Neurosci. 27, 2152–2166. 10.1038/s41593-024-01767-4 39284963 PMC11932732

[B115] McGuire J. T. Botvinick M. M. (2010). Prefrontal cortex, cognitive control, and the registration of decision costs. Proc. Natl. Acad. Sci. U. S. A. 107, 7922–7926. 10.1073/pnas.0910662107 20385798 PMC2867898

[B116] McIntire L. McKinley A. Key M. (2023). Cervical transcutaneous vagal nerve stimulation to improve mission qualification training for an AFSOC full motion video/geospatial analysis squadron. Brain Stimul. Basic, Transl. Clin. Res. Neuromodulation 16, 229. 10.1016/j.brs.2023.01.338

[B117] McIntire L. K. McKinley R. A. Goodyear C. McIntire J. P. Brown R. D. (2021). Cervical transcutaneous vagal nerve stimulation (ctVNS) improves human cognitive performance under sleep deprivation stress. Commun. Biol. 4, 634. 10.1038/s42003-021-02145-7 34112935 PMC8192899

[B118] McIntire L. K. McKinley R. A. Goodyear C. Nelson J. (2014). A comparison of the effects of transcranial direct current stimulation and caffeine on vigilance and cognitive performance during extended wakefulness. Brain Stimul. 7, 499–507. 10.1016/j.brs.2014.04.008 25047826

[B119] McKinley R. A. McIntire L. Bridges N. Goodyear C. Bangera N. B. Weisend M. P. (2013). Acceleration of image analyst training with transcranial direct current stimulation. Behav. Neurosci. 127, 936–946. 10.1037/a0034975 24341718

[B120] McKinley R. A. McIntire L. K. Funke M. A. (2011). Operator selection for unmanned aerial systems: comparing video game players and pilots. Aviat. Space, Environ. Med. 82, 635–642. 10.3357/asem.2958.2011 21702315

[B121] Meads K. L. Huettner S. Amata D. Johnson H. Devine J. K. Warnakulasuriya S. (2024). Feasibility and acceptability of wearing a neuromodulation device at night in individuals in recovery from opioid use disorder. Front. Psychiatry 15, 1481795. 10.3389/fpsyt.2024.1481795 39676914 PMC11640868

[B122] Merkert R. Bushell J. (2020). Managing the drone revolution: a systematic literature review into the current use of airborne drones and future strategic directions for their effective control. J. Air Transp. Manag. 89, 101929. 10.1016/j.jairtraman.2020.101929 32952321 PMC7489224

[B123] Mirzaei M. Kan P. Kaufmann H. (2020). EarVR: using ear haptics in virtual reality for deaf and hard-of-hearing people. IEEE Trans. Vis. Comput. Graph 26, 2084–2093. 10.1109/tvcg.2020.2973441 32070977

[B124] Miyatsu T. Oviedo V. Reynaga J. Karuzis V. P. Martinez D. O’Rourke P. (2024). Transcutaneous cervical vagus nerve stimulation enhances second-language vocabulary acquisition while simultaneously mitigating fatigue and promoting focus. Sci. Rep. 14, 17177. 10.1038/s41598-024-68015-4 39060415 PMC11282064

[B125] Moazzami K. Pearce B. D. Gurel N. Z. Wittbrodt M. T. Levantsevych O. M. Huang M. (2023). Transcutaneous vagal nerve stimulation modulates stress-induced plasma ghrelin levels: a double-blind, randomized, sham-controlled trial. J. Affect. Disord. 342, 85–90. 10.1016/j.jad.2023.09.015 37714385 PMC10698687

[B126] Moit H. Dwyer A. De Sutter M. Heinzel S. Crawford D. (2019). A standardized robotic training curriculum in a general surgery program. Jsls 23, e2019.00045. 10.4293/jsls.2019.00045 PMC6924504 31892790

[B127] Muller-Putz G. R. Scherer R. Neuper C. Pfurtscheller G. (2006). Steady-state somatosensory evoked potentials: suitable brain signals for brain-computer interfaces? IEEE Trans. Neural Syst. Rehabilitation Eng. 14, 30–37. 10.1109/tnsre.2005.863842 16562629

[B128] Naor O. Krupa S. Shoham S. (2016). Ultrasonic neuromodulation. J. Neural Eng. 13, 031003. 10.1088/1741-2560/13/3/031003 27153566

[B129] Nejati V. Mirikaram F. Nitsche M. A. (2024). Transcranial direct current stimulation improves time perception in children with ADHD. Sci. Rep. 14, 31807. 10.1038/s41598-024-82974-8 39738488 PMC11686130

[B130] Nejati V. Movahed Alavi M. Nitsche M. A. (2021). The impact of attention deficit-hyperactivity disorder symptom severity on the effectiveness of transcranial direct current stimulation (tDCS) on inhibitory control. Neuroscience 466, 248–257. 10.1016/j.neuroscience.2021.05.008 34015371

[B131] Nejati V. Salehinejad M. A. Nitsche M. A. Najian A. Javadi A.-H. (2020). Transcranial direct current stimulation improves executive dysfunctions in ADHD: implications for inhibitory control, interference control, working memory, and cognitive flexibility. J. Atten. Disord. 24, 1928–1943. 10.1177/1087054717730611 28938852

[B132] Nelson J. McKinley R. A. Phillips C. McIntire L. Goodyear C. Kreiner A. (2016). The effects of transcranial direct current stimulation (tDCS) on multitasking throughput capacity. Front. Hum. Neurosci. 10, 589. 10.3389/fnhum.2016.00589 27965553 PMC5126079

[B133] Nelson J. T. McKinley R. A. Golob E. J. Warm J. S. Parasuraman R. (2014). Enhancing vigilance in operators with prefrontal cortex transcranial direct current stimulation (tDCS). Neuroimage 85 (Pt 3), 909–917. 10.1016/j.neuroimage.2012.11.061 23235272

[B134] Neri F. Della Toffola J. Scoccia A. Benelli A. Lomi F. Cinti A. (2025). Neuromodulation via tRNS accelerates learning and enhances in-game performance at a virtual-reality first person shooter game. Comput. Hum. Behav. 165, 108537. 10.1016/j.chb.2024.108537

[B135] Neuser M. P. Teckentrup V. Kühnel A. Hallschmid M. Walter M. Kroemer N. B. (2020). Vagus nerve stimulation boosts the drive to work for rewards. Nat. Commun. 11, 3555. 10.1038/s41467-020-17344-9 32678082 PMC7366927

[B136] Nitsche M. A. Paulus W. (2000). Excitability changes induced in the human motor cortex by weak transcranial direct current stimulation. J. Physiol. 527 (Pt 3), 633–639. 10.1111/j.1469-7793.2000.t01-1-00633.x 10990547 PMC2270099

[B137] Olsen L. K. Solis E. McIntire L. K. Hatcher-Solis C. N. (2023). Vagus nerve stimulation: mechanisms and factors involved in memory enhancement. Front. Hum. Neurosci. 17, 1152064. 10.3389/fnhum.2023.1152064 37457500 PMC10342206

[B138] Orban de Xivry J.-J. Shadmehr R. (2014). Electrifying the motor engram: effects of tDCS on motor learning and control. Exp. Brain Res. 232, 3379–3395. 10.1007/s00221-014-4087-6 25200178 PMC4199902

[B139] Ouellet J. McGirr A. Van den Eynde F. Jollant F. Lepage M. Berlim M. T. (2015). Enhancing decision-making and cognitive impulse control with transcranial direct current stimulation (tDCS) applied over the orbitofrontal cortex (OFC): a randomized and sham-controlled exploratory study. J. Psychiatric Res. 69, 27–34. 10.1016/j.jpsychires.2015.07.018 26343591

[B140] Pandža N. B. Phillips I. Karuzis V. P. O'Rourke P. Kuchinsky S. E. (2020). Neurostimulation and pupillometry: new directions for learning and research in applied linguistics. Annu. Rev. Appl. Linguistics 40, 56–77. 10.1017/s0267190520000069

[B141] Patel R. V. Atashzar S. F. Tavakoli M. (2022). Haptic feedback and force-based teleoperation in surgical robotics. Proc. IEEE 110, 1012–1027. 10.1109/jproc.2022.3180052

[B142] Paulus W. (2011). Transcranial electrical stimulation (tES - tDCS; tRNS, tACS) methods. Neuropsychol. Rehabil. 21, 602–617. 10.1080/09602011.2011.557292 21819181

[B143] Pavlov V. A. Tracey K. J. (2005). The cholinergic anti-inflammatory pathway. Brain Behav. Immun. 19, 493–499. 10.1016/j.bbi.2005.03.015 15922555

[B144] Phillips I. Calloway R. C. Karuzis V. P. Pandža N. B. O'Rourke P. Kuchinsky S. E. (2021). Transcutaneous auricular vagus nerve stimulation strengthens semantic representations of foreign language tone words during initial stages of learning. J. Cognitive Neurosci. 34, 127–152. 10.1162/jocn_a_01783 34673939

[B145] Pihlaja M. Failla L. Peräkylä J. Hartikainen K. M. (2020). Reduced frontal nogo-N2 with uncompromised response inhibition during transcutaneous vagus nerve stimulation—more efficient cognitive control? Front. Hum. Neurosci. 14, 561780. 10.3389/fnhum.2020.561780 33132877 PMC7573492

[B146] Pizoń J. Gola A. (2023). Human–machine relationship—perspective and future roadmap for industry 5.0 solutions. Machines 11, 203. 10.3390/machines11020203

[B147] Prescott S. L. Liberles S. D. (2022). Internal senses of the vagus nerve. Neuron 110, 579–599. 10.1016/j.neuron.2021.12.020 35051375 PMC8857038

[B148] Ramakrishnan P. Balasingam B. Biondi F. (2021). “Chapter 2 - cognitive load estimation for adaptive human–machine system automation,” in Learning control. Editors Zhang D. Wei B. (Elsevier), 35–58.

[B149] Rao B. Gopi A. G. Maione R. (2016). The societal impact of commercial drones. Technol. Soc. 45, 83–90. 10.1016/j.techsoc.2016.02.009

[B150] Ridgewell C. Heaton K. J. Hildebrandt A. Couse J. Leeder T. Neumeier W. H. (2021). The effects of transcutaneous auricular vagal nerve stimulation on cognition in healthy individuals: a meta-analysis. Neuropsychology 35, 352–365. 10.1037/neu0000735 34043386

[B151] Rossi S. Antal A. Bestmann S. Bikson M. Brewer C. Brockmöller J. (2021). Safety and recommendations for TMS use in healthy subjects and patient populations, with updates on training, ethical and regulatory issues: expert Guidelines. Clin. Neurophysiol. 132, 269–306. 10.1016/j.clinph.2020.10.003 33243615 PMC9094636

[B152] Ruf S. P. Fallgatter A. J. Plewnia C. (2017). Augmentation of working memory training by transcranial direct current stimulation (tDCS). Sci. Rep. 7, 876. 10.1038/s41598-017-01055-1 28432349 PMC5430723

[B153] Rufener K. S. Geyer U. Janitzky K. Heinze H. J. Zaehle T. (2018). Modulating auditory selective attention by non-invasive brain stimulation: differential effects of transcutaneous vagal nerve stimulation and transcranial random noise stimulation. Eur. J. Neurosci. 48, 2301–2309. 10.1111/ejn.14128 30144194

[B154] Ruhnau P. Zaehle T. (2021). Transcranial auricular vagus nerve stimulation (taVNS) and ear-EEG: potential for closed-loop portable non-invasive brain stimulation. Front. Hum. Neurosci. 15, 699473. 10.3389/fnhum.2021.699473 34194308 PMC8236702

[B155] Sakib M. N. Chaspari T. Behzadan A. H. (2021). Physiological data models to understand the effectiveness of drone operation training in immersive virtual reality. J. Comput. Civ. Eng. 35, 04020053. 10.1061/(asce)cp.1943-5487.0000941

[B156] Sant'Anna F. M. Resende R. C. L. Sant'Anna L. B. Couceiro S. L. M. Pinto R. B. S. Sant'Anna M. B. (1992). Auricular vagus nerve stimulation: a new option to treat inflammation in COVID-19? Rev. Assoc. Med. Bras. 69, e20230345. 10.1590/1806-9282.20230345 PMC10241082 37283364

[B157] Santarnecchi E. Brem A.-K. Levenbaum E. Thompson T. Kadosh R. C. Pascual-Leone A. (2015). Enhancing cognition using transcranial electrical stimulation. Curr. Opin. Behav. Sci. 4, 171–178. 10.1016/j.cobeha.2015.06.003

[B158] Saucedo Marquez C. M. Zhang X. Swinnen S. P. Meesen R. Wenderoth N. (2013). Task-specific effect of transcranial direct current stimulation on motor learning. Front. Hum. Neurosci. 7, 333. 10.3389/fnhum.2013.00333 23847505 PMC3696911

[B159] Schmidt R. Schadow J. Eißfeldt H. Pecena Y. (2022). Insights on remote pilot competences and training needs of civil drone pilots. Transp. Res. Procedia 66, 1–7. 10.1016/j.trpro.2022.12.001

[B160] Schönböck J. Kurschl W. Augstein M. Altmann J. Fraundorfer J. Freller L.-M. (2022). From remote-controlled excavators to digitized construction sites. Procedia Comput. Sci. 200, 1155–1164. 10.1016/j.procs.2022.01.315

[B161] Schuerman W. L. Nourski K. V. Rhone A. E. Howard M. A. Chang E. F. Leonard M. K. (2021). Human intracranial recordings reveal distinct cortical activity patterns during invasive and non-invasive vagus nerve stimulation. Sci. Rep. 11, 22780. 10.1038/s41598-021-02307-x 34815529 PMC8611055

[B162] Sharon O. Fahoum F. Nir Y. (2021). Transcutaneous vagus nerve stimulation in humans induces pupil dilation and attenuates alpha oscillations. J. Neurosci. Official J. Soc. Neurosci. 41, 320–330. 10.1523/jneurosci.1361-20.2020 PMC7810665 33214317

[B163] Shringi A. Arashpour M. Golafshani E. M. Rajabifard A. Dwyer T. Li H. (2022). Efficiency of VR-based safety training for construction equipment: hazard recognition in heavy machinery operations. Buildings 12, 2084. 10.3390/buildings12122084

[B164] Somerville A. Lynar T. Joiner K. Wild G. (2024). Use of simulation for pre-training of drone pilots. Drones 8, 640. 10.3390/drones8110640

[B165] Sommer A. Fischer R. Borges U. Laborde S. Achtzehn S. Liepelt R. (2023). The effect of transcutaneous auricular vagus nerve stimulation (taVNS) on cognitive control in multitasking. Neuropsychologia 187, 108614. 10.1016/j.neuropsychologia.2023.108614 37295553

[B166] Soori M. Arezoo B. Dastres R. (2023). Artificial intelligence, machine learning and deep learning in advanced robotics, a review. Cogn. Robot. 3, 54–70. 10.1016/j.cogr.2023.04.001

[B167] Souza R. H. C. e. Naves E. L. M. (2021). Attention detection in virtual environments using EEG signals: a scoping review. Front. Physiology 12, 727840. 10.3389/fphys.2021.727840 PMC8650681 34887770

[B168] Soyata A. Z. Aksu S. Woods A. J. İşçen P. Saçar K. T. Karamürsel S. (2019). Effect of transcranial direct current stimulation on decision making and cognitive flexibility in gambling disorder. Eur. Arch. Psychiatry Clin. Neurosci. 269, 275–284. 10.1007/s00406-018-0948-5 30367243

[B169] Sridhar A. N. Briggs T. P. Kelly J. D. Nathan S. (2017). Training in robotic surgery—an overview. Curr. Urol. Rep. 18, 58. 10.1007/s11934-017-0710-y 28647793 PMC5486586

[B170] Srinivasan V. Abathsagayam K. Suganthirababu P. Alagesan J. Vishnuram S. Vasanthi R. K. (2023). Effect of vagus nerve stimulation (taVNS) on anxiety and sleep disturbances among elderly health care workers in the post COVID-19 pandemic. Work 78, 1149–1156. 10.3233/wor-231362 38143418

[B171] Su S. Chai G. Shu X. Sheng X. Zhu X. (2020). Electrical stimulation-induced SSSEP as an objective index to evaluate the difference of tactile acuity between the left and right hand. J. Neural Eng. 17, 016053. 10.1088/1741-2552/ab5ee9 31801122

[B172] Sun J.-B. Cheng C. Tian Q.-Q. Yuan H. Yang X.-J. Deng H. (2021). Transcutaneous auricular vagus nerve stimulation improves spatial working memory in healthy young adults. Front. Neurosci. 15, 790793. 10.3389/fnins.2021.790793 35002607 PMC8733384

[B173] Tan C. Qiao M. Ma Y. Luo Y. Fang J. Yang Y. (2023). The efficacy and safety of transcutaneous auricular vagus nerve stimulation in the treatment of depressive disorder: a systematic review and meta-analysis of randomized controlled trials. J. Affect. Disord. 337, 37–49. 10.1016/j.jad.2023.05.048 37230264

[B174] Tezza D. Andujar M. (2019). The state-of-the-art of human–drone interaction: a survey. IEEE Access 7, 167438–167454. 10.1109/access.2019.2953900

[B175] Thakur S. Armas N. D. Adegite J. Pandey R. Mead J. Rao P. M. (2024). A tetherless soft robotic wearable haptic human machine interface for robot teleoperation. IEEE/RSJ International Conference on Intelligent Robots and Systems IROS, 12226–12233.

[B176] Thayer J. F. Sternberg E. (2006). Beyond heart rate variability: vagal regulation of allostatic systems. Ann. N. Y. Acad. Sci. 1088, 361–372. 10.1196/annals.1366.014 17192580

[B177] Tonin L. Bauer F. C. Millán J. del R. (2020). The role of the control framework for continuous teleoperation of a brain–machine interface-driven mobile robot. IEEE Trans. Robotics 36, 78–91. 10.1109/tro.2019.2943072

[B178] Triantafyllidis E. Mcgreavy C. Gu J. Li Z. (2020). Study of multimodal interfaces and the improvements on teleoperation. IEEE Access 8, 78213–78227. 10.1109/access.2020.2990080

[B179] Trifilio E. Shortell D. Olshan S. O’Neal A. Coyne J. Lamb D. (2023). Impact of transcutaneous vagus nerve stimulation on healthy cognitive and brain aging. Front. Neurosci. 17, 1184051. 10.3389/fnins.2023.1184051 37575296 PMC10416636

[B180] Tufail Y. Matyushov A. Baldwin N. Tauchmann M. L. Georges J. Yoshihiro A. (2010). Transcranial pulsed ultrasound stimulates intact brain circuits. Neuron 66, 681–694. 10.1016/j.neuron.2010.05.008 20547127

[B181] Tyler W. Sanguinetti J. Fini M. Hool N. (2017). Non-invasive neural stimulation, 10194, 101941L, 10.1117/12.2263175

[B182] Tyler W. J. (2017). Multimodal neural interfaces for augmenting human cognition. Cham: Springer International Publishing, 389–407.

[B183] Tyler W. J. (2025). Auricular bioelectronic devices for health, medicine, and human-computer interfaces. Front. Electron. 6. 10.3389/felec.2025.1503425

[B184] Tyler W. J. Lani S. W. Hwang G. M. (2018). Ultrasonic modulation of neural circuit activity. Curr. Opin. Neurobiol. 50, 222–231. 10.1016/j.conb.2018.04.011 29674264

[B185] Tyler W. J. Tufail Y. Finsterwald M. Tauchmann M. L. Olson E. J. Majestic C. (2008). Remote excitation of neuronal circuits using low-intensity, low-frequency ultrasound. PLoS One 3, e3511. 10.1371/journal.pone.0003511 18958151 PMC2568804

[B186] Tyler W. J. Wyckoff S. Hearn T. Hool N. (2019). The safety and efficacy of transdermal auricular vagal nerve stimulation earbud electrodes for modulating autonomic arousal, attention, sensory gating, and cortical brain plasticity in humans. bioRxiv, 732529. 10.1101/732529

[B187] Urbin M. A. Lafe C. W. Simpson T. W. Wittenberg G. F. Chandrasekaran B. Weber D. J. (2021). Electrical stimulation of the external ear acutely activates noradrenergic mechanisms in humans. Brain Stimul. 14, 990–1001. 10.1016/j.brs.2021.06.002 34154980

[B188] Veitch E. Andreas Alsos O. (2022). A systematic review of human-AI interaction in autonomous ship systems. Saf. Sci. 152, 105778. 10.1016/j.ssci.2022.105778

[B189] Verma N. Mudge J. D. Kasole M. Chen R. C. Blanz S. L. Trevathan J. K. (2021). Auricular vagus neuromodulation—a systematic review on quality of evidence and clinical effects. Front. Neurosci. 15, 664740. 10.3389/fnins.2021.664740 33994937 PMC8120162

[B190] Wanasinghe T. R. Trinh T. Nguyen T. Gosine R. G. James L. A. Warrian P. J. (2021). Human centric digital transformation and operator 4.0 for the oil and gas industry. IEEE Access 9, 113270–113291. 10.1109/access.2021.3103680

[B191] Wang W. Jiang Y. Zhong D. Zhang Z. Choudhury S. Lai J.-C. (2023). Neuromorphic sensorimotor loop embodied by monolithically integrated, low-voltage, soft e-skin. Science 380, 735–742. 10.1126/science.ade0086 37200416

[B192] Wang Y. Li S. Y. Wang D. Wu M. Z. He J. K. Zhang J. L. (2021). Transcutaneous auricular vagus nerve stimulation: from concept to application. Neurosci. Bull. 37, 853–862. 10.1007/s12264-020-00619-y 33355897 PMC8192665

[B193] Warren A. D. Proctor R. W. Dunston P. S. (2023). Effect of motion feedback on skill acquisition: training performance using an excavator simulator. Proc. Hum. Factors Ergonomics Soc. Annu. Meet. 67, 1329–1330. 10.1177/21695067231192701

[B194] Xia P. Xu F. Song Z. Li S. Du J. (2023). Sensory augmentation for subsea robot teleoperation. Comput. Industry 145, 103836. 10.1016/j.compind.2022.103836

[B195] Xu C. Li B. Xu C. Zheng J. (2015). A novel dielectric elastomer actuator based on compliant polyvinyl alcohol hydrogel electrodes. J. Mater. Sci. Mater. Electron. 26, 9213–9218. 10.1007/s10854-015-3614-y

[B196] Xu Z. Zheng N. (2021). Incorporating virtual reality technology in safety training solution for construction site of urban cities. Sustainability 13, 243. 10.3390/su13010243

[B197] Yang T.-H. Kim J. R. Jin H. Gil H. Koo J.-H. Kim H. J. (2021). Recent advances and opportunities of active materials for haptic technologies in virtual and augmented reality. Adv. Funct. Mater. 31, 2008831. 10.1002/adfm.202008831

[B198] Yang X. Gao M. Shi J. Ye H. Chen S. (2017). Modulating the activity of the dlpfc and OFC has distinct effects on risk and ambiguity decision-making: a tDCS study. Front. Psychol. 8, 1417. 10.3389/fpsyg.2017.01417 28878714 PMC5572270

[B199] Yin J. Hinchet R. Shea H. Majidi C. (2021). Wearable soft technologies for haptic sensing and feedback. Adv. Funct. Mater. 31, 2007428. 10.1002/adfm.202007428

[B200] Zafar A. Ghafoor U. Khan M. J. Hong K. S. (2018). “Drone control using functional near-infrared spectroscopy,” in 2018 15th international conference on electrical engineering/electronics, computer, telecommunications and information technology (ECTI-CON), 384–387.

[B201] Zhang Y. Zhao J. h. Huang D. y. Chen W. Yuan C. x. Jin L. r. (2020). Multiple comorbid sleep disorders adversely affect quality of life in Parkinson’s disease patients. npj Parkinson's Dis. 6, 25–27. 10.1038/s41531-020-00126-x 33015354 PMC7492275

[B202] Zhao R. Chang M. Y. Cheng C. Tian Q. Q. Yang X. J. Du M. Y. (2023). Transcutaneous auricular vagus stimulation (taVNS) improves human working memory performance under sleep deprivation stress. Behav. Brain Res. 439, 114247. 10.1016/j.bbr.2022.114247 36473677

[B203] Zhu Q. Du J. Shi Y. Wei P. (2021). Neurobehavioral assessment of force feedback simulation in industrial robotic teleoperation. Automation Constr. 126, 103674. 10.1016/j.autcon.2021.103674

